# Control of contextual memory through interneuronal α5-GABA_A_ receptors

**DOI:** 10.1093/pnasnexus/pgad065

**Published:** 2023-04-11

**Authors:** Mengwen Zhu, Alifayaz Abdulzahir, Mark G Perkins, Chan C Chu, Bryan M Krause, Cameron Casey, Richard Lennertz, David Ruhl, Harald Hentschke, Rajasekar Nagarajan, Edwin R Chapman, Uwe Rudolph, Michael S Fanselow, Robert A Pearce

**Affiliations:** Department of Anesthesiology, University of Wisconsin-Madison, Madison, WI 53705, USA; Department of Anesthesiology, University of Wisconsin-Madison, Madison, WI 53705, USA; Department of Anesthesiology, University of Wisconsin-Madison, Madison, WI 53705, USA; Department of Anesthesiology, University of Wisconsin-Madison, Madison, WI 53705, USA; Present address: Lawrence University, Appleton, WI 54911, USA; Department of Anesthesiology, University of Wisconsin-Madison, Madison, WI 53705, USA; Department of Anesthesiology, University of Wisconsin-Madison, Madison, WI 53705, USA; Department of Anesthesiology, University of Wisconsin-Madison, Madison, WI 53705, USA; Department of Neuroscience, University of Wisconsin-Madison, Madison, WI 53705, USA; Present address: Neurocrine Biosciences, San Diego, CA 92130, USA; Freelance Data Scientist, Berlin 12555, Germany; Department of Comparative Biosciences, University of Illinois at Urbana-Champaign, Urbana, IL 61802, USA; Department of Neuroscience, University of Wisconsin-Madison, Madison, WI 53705, USA; Department of Comparative Biosciences, University of Illinois at Urbana-Champaign, Urbana, IL 61802, USA; Carl R. Woese Institute for Genomic Biology, University of Illinois at Urbana-Champaign, Urbana, IL 61802, USA; Department of Psychology, University of California, Los Angeles, Los Angeles, CA 61801, USA; Department of Psychiatry, University of California, Los Angeles, Los Angeles, CA 90095, USA; Department of Anesthesiology, University of Wisconsin-Madison, Madison, WI 53705, USA

## Abstract

γ-Aminobutyric acid type A receptors that incorporate α5 subunits (α5-GABA_A_Rs) are highly enriched in the hippocampus and are strongly implicated in control of learning and memory. Receptors located on pyramidal neuron dendrites have long been considered responsible, but here we report that mice in which α5-GABA_A_Rs have been eliminated from pyramidal neurons (α5-pyr-KO) continue to form strong spatial engrams and that they remain as sensitive as their pseudo-wild-type (p-WT) littermates to etomidate-induced suppression of place cells and spatial engrams. By contrast, mice with selective knockout in interneurons (α5-i-KO) no longer exhibit etomidate-induced suppression of place cells. In addition, the strength of spatial engrams is lower in α5-i-KO mice than p-WT littermates under control conditions. Consistent with the established role of the hippocampus in contextual fear conditioning, α5-i-KO mice resisted etomidate’s suppression of freezing to context, but so too did α5-pyr-KO mice, supporting a role for extra-hippocampal regions in the development of contextual fear memory. Overall, our results indicate that interneuronal α5-GABA_A_Rs serve a physiological role in promoting spatial learning and that they mediate suppression of hippocampus-dependent contextual memory by etomidate.

**Significance Statement**

Etomidate is a prototypical general anesthetic that potently blocks memory formation even at subanesthetic doses by targeting GABA_A_Rs that incorporate α5 subunits (α5-GABA_A_Rs). These receptors are highly enriched in the hippocampus, a brain structure that is essential for the formation of episodic memories. α5-GABA_A_Rs located on pyramidal neuron dendrites have long been considered responsible, but here we report that selectively eliminating them from interneurons prevents etomidate from blocking hippocampus-dependent memory and from suppressing the spatial/cognitive map formed within the hippocampus. Given the well-established role of α5-GABA_A_Rs in cognitive function, the findings may have application to diverse disease states including Alzheimer's disease, Down syndrome, autism, depression, and schizophrenia.

## Introduction

Inhibitory synaptic transmission in the brain is mediated primarily by γ-aminobutyric acid type A receptors (GABA_A_Rs). This family of chloride-permeable ionotropic receptors is the target of a wide variety of clinically important agents, including sedative hypnotics, anxiolytics, anticonvulsants, and general anesthetics (1, 2). Many of these agents also impair memory. Usually, this is an undesired side effect, but for general anesthetics, it is a primary goal. Indeed, awareness with recall during anesthesia is recognized as a persistent problem (3–5), and it has been associated with dysphoria and post-traumatic stress disorder (4, 6). In studies of the mechanism of anesthetic-induced amnesia, etomidate has proved exceptionally useful, in part not only because it is highly selective for GABA_A_ receptors but also because its physicochemical properties make it amenable to use both in vivo and in vitro (7, 8).

Etomidate potently blocks memory formation even at subanesthetic doses by targeting GABA_A_Rs that incorporate α5 subunits (α5-GABA_A_Rs) (9, 10). These receptors are expressed at intermediate levels throughout the cortex, including frontal and prefrontal cortex (11, 12), but they are most highly concentrated in the hippocampus (13, 14), a brain structure that is essential for the formation of episodic memories (15). The well-established role of α5-GABA_A_Rs in learning and memory has prompted the development of agents that target α5-GABA_A_Rs to enhance cognition (16–20), with application to diverse disease states including Alzheimer's disease, Down syndrome, autism, depression, and schizophrenia (18, 21–28).

The generally accepted mechanism by which α5-GABA_A_Rs modulate memory involves receptors on pyramidal neurons (29–31). These receptors are located primarily at extrasynaptic sites on pyramidal neuron dendrites (32), and acting through either tonic (33) or slow phasic inhibition (34), α5-GABA_A_Rs are well suited to prevent the depolarization and NMDAR-mediated calcium entry that initiates synaptic plasticity. Accordingly, GABAergic drugs such as etomidate that are normally able to suppress long-term potentiation (LTP) and learning are ineffective in mice with a global knockout of α5 subunits (α5-gl-KO) or in mice administered α5-selective negative modulators (9, 10). However, recent studies indicate that α5-GABA_A_Rs are not restricted to pyramidal neurons (35, 36), raising the possibility that interneuronal α5-GABA_A_Rs might also play a role. Indeed, we found that selective knockout of α5 subunits from pyramidal neurons did not recapitulate the resistance to LTP suppression seen in α5-gl-KO mice (37) and that suppression of LTP and contextual fear conditioning were independent of GABA_A_Rs that incorporate β3 subunits, which underlie tonic and long-lasting phasic inhibition in pyramidal neurons (38, 39). These findings raised the possibility that α5-GABA_A_Rs on pyramidal neurons might not be the only targets mediating etomidate-induced amnesia. In the present study, we therefore tested the contributions of α5-GABA_A_Rs on interneurons versus pyramidal neurons by studying the effects of etomidate on contextual fear conditioning as well as place cells and spatial engrams as neural correlates of spatial memory, in interneuron-selective α5-GABA_A_R knockout (α5-i-KO), pyramidal neuron-selective α5-GABA_A_R knockout (α5-pyr-KO) mice, and pseudo-wild-type (p-WT) mice.

## Results

### Cell type-specific elimination of α5-GABA_A_Rs

**Fig. 1.**
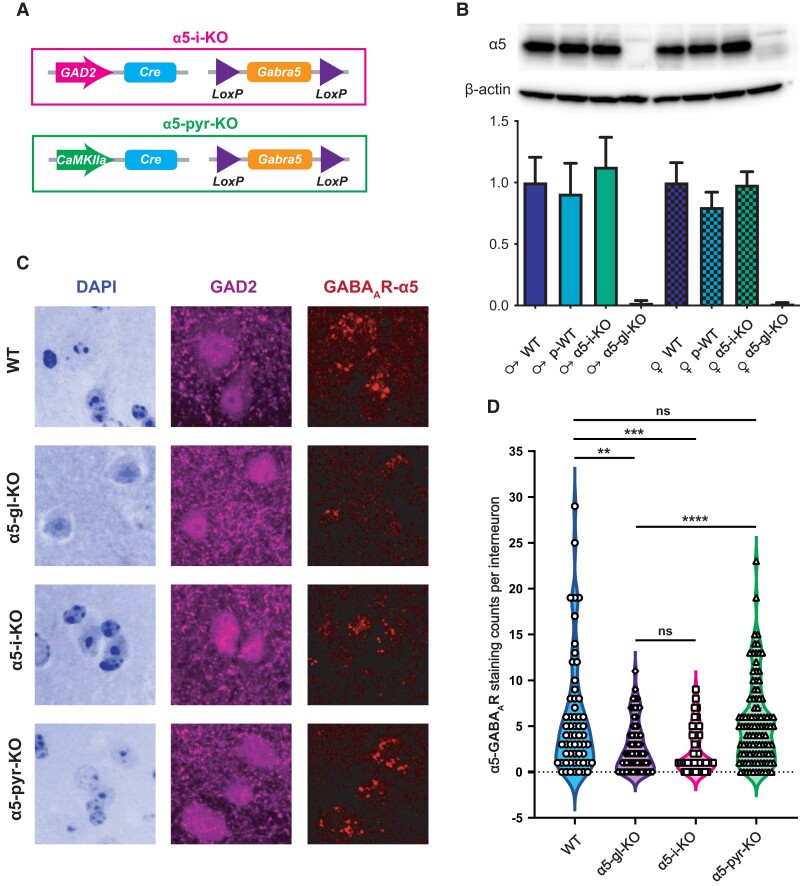
Specific elimination of α5-GABA_A_Rs from interneurons versus pyramidal neurons. A) Genetic strategies used to eliminate α5-GABA_A_Rs from interneurons (top) or pyramidal neurons (bottom). For α5-i-KO mice, Cre recombinase was driven by the interneuron-specific GAD2 promoter to target the floxed Gabra5 gene. For α5-pyr-KO mice, Cre was driven by the pyramidal neuron-specific CaMKIIα promoter. B) Western blot analysis of α5-GABA_A_R (55 kDa) expression in male and female WT, p-WT, α5-i-KO, and α5-gl-KO mice. Expression levels were normalized to β-actin (42 kDa) and quantified by densitometry. Note that receptor levels were unchanged in p-WT and α5-i-KO mice versus WT mice, consistent with maintained expression in pyramidal neurons. C) Immunohistochemical analysis of α5-GABA_A_R expression levels in interneurons, in WT, α5-gl-KO, α5-i-KO, and α5-pyr-KO mice (ordered from top to bottom). Significant loss of α5-GABA_A_R staining from interneurons (GAD2-positive cells) was seen in α5-gl-KO and α5-i-KO but not α5-pyr-KO mice. (D) Quantification of punctate α5-GABA_A_R staining within all identified interneurons (GAD2+/DAPI+ neurons) in WT, α5-gl-KO, α5-i-KO, and α5-pyr-KO mice (*N* = 3 for each genotype). Expression levels were significantly reduced in α5-gl-KO and α5-i-KO mice but not in α5-pyr-KO mice.

To generate mice lacking α5-GABA_A_Rs specifically in interneurons or pyramidal neurons, we crossed mice carrying a floxed α5 allele (Gabra5^tm2.1Uru^) with mice that express Cre recombinase under the control of the GAD2 promoter (Gad2^tm2(Cre)Zjh^) or CaMKIIα promoter (Tg(Camk2a-Cre)T29-1Stl), respectively (Fig. 1A). We showed previously by immunohistochemistry (IHC) that α5-GABA_A_Rs in hippocampus are strongly reduced in α5-pyr-KO mice, to a level comparable to that of global α5-GABA_A_R knockout mice (α5-gl-KO), consistent with a predominance of α5-GABA_A_Rs in pyramidal neurons (37). Western blot analysis of α5-i-KO mice similarly showed that p-WT mice and α5-i-KO mice expressed α5 subunits at levels indistinguishable from C57BL/6J (WT) mice (Fig. 1B), again consistent with predominant and maintained expression of α5-GABA_A_Rs in pyramidal neurons. To test whether the GAD2-Cre promoter might lead to loss of α5-GABA_A_Rs in astrocytes, which also express GABA_A_Rs and synthesize and release GABA (40), we tested for co-expression of the GAD2-Cre-driven tdTomato reporter and GFAP-driven green fluorescent protein (GFP) (Figure S1A). We found complete segregation of the markers (Fig. S1B), further supporting the selective knockout of α5 subunits from interneurons but not glia in α5-i-KO mice. Finally, to directly demonstrate a reduction in α5-GABA_A_R protein in interneurons, we examined co-localization of α5 subunits and GAD2 in WT, α5-gl-KO, α5-i-KO, and α5-pyr-KO mice. We found that α5-GABA_A_Rs and GAD2 were co-localized on interneurons in all layers in WT and α5-pyr-KO mice, but co-localized markers were significantly lower in α5-gl-KO and α5-i-KO mice (Fig. 1C). Quantification of α5-GABA_A_R staining within GAD2+/DAPI+ neurons (275 cells) based on 51 Z-stack images taken from three mice of each genotype confirmed significant reductions of α5-GABA_A_R expression in α5-gl-KO (*U* = 1362, *P* = 0.0023) and α5-i-KO (*U* = 852, *P* = 0.0009) mice compared to WT mice (Fig. 1D). Expression levels were not reduced in α5-pyr-KO mice (*U* = 2969, *P* = 0.8093). Residual expression in α5-gl-KO and α5-i-KO mice presumably reflects a combination of background nonspecific plus off-target (e.g. other GABA_A_R α-subunit) staining. Together, these findings demonstrate that α5-GABA_A_Rs were successfully eliminated in a selective fashion in α5-i-KO and α5-pyr-KO mice.

### α5-i-KO and α5-pyr-KO mice resist the suppression of contextual conditioning by etomidate

**Fig. 2.**
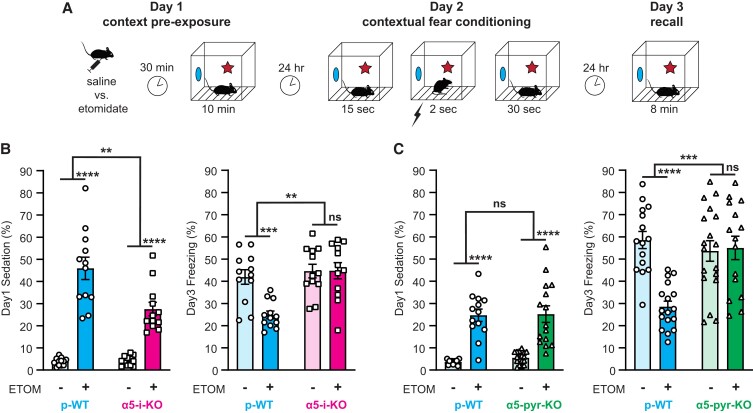
α5-i-KO and α5-pyr-KO mice resist the suppression of contextual learning by etomidate. A) Context preexposure facilitation effect (CPFE) paradigm. B) Effect of etomidate in α5-i-KO and p-WT mice. Etomidate (7 mg/kg IP) sedated both gentoypes, p-WT more than α5-i-KO mice (left), but impaired memory in only p-WT mice (right). *P < 0.05, **P < 0.01, ***P < 0.001, ****P < 0.0001. C) Effect of etomidate in α5-pyr-KO and p-WT mice. Etomidate sedated both gentoypes equally but impaired memory in only p-WT mice (right). *P < 0.05, **P < 0.01, ***P < 0.001, ****P < 0.0001.

To test the role of α5-GABA_A_Rs expressed on interneurons and pyramidal neurons in etomidate's suppression of hippocampus-dependent memory, we employed the context preexposure facilitation effect (CPFE) learning paradigm (Fig. 2A)—a variant of contextual fear conditioning that separates the contextual learning and aversive phases of conditioning (41–44). This paradigm takes advantage of the “immediate shock deficit” (45), wherein mice that are shocked immediately after they are placed in a novel context fail to associate the context and shock, whereas mice that were preexposed to the context on the preceding day recall the context rapidly and successfully associate it with the shock (46, 47). Because the drug is present only during contextual learning (day 1) and not when the shock is administered (day 2), any observed change in freezing behavior (day 3) can be attributed to a failure to learn the context rather than an attenuation of the shock's effectiveness.

There were no baseline differences between α5-i-KO mice and their p-WT littermates (p-WT_i_) in anxiety, as indicated by open arm entries or open arm time in the elevated plus maze test, or in pain sensitivity, as indicated by time to withdrawal in the hot plate test (Fig. S2). Etomidate (7 mg/kg IP, 30 min before day 1) did produce a greater level of sedation (day 1) in p-WT_i_ compared to α5-i-KO mice [Fig. 2B, *left*; drug × genotype, *F*(1, 44) = 10.04, *P* = 0.0028], but on average, both genotypes spent more than one-half of their time actively exploring the novel context during the context preexposure. Most importantly, etomidate differentially suppressed freezing (day 3) in α5-i-KO versus p-WT_i_ mice [Fig. 2B, *right*; drug × genotype, *F*(1, 44) = 8.09, *P* = 0.0067], with a significant suppression of freezing in p-WT_i_ mice [*t*(44) = 3.96, *P* < 0.001] but not in α5-i-KO mice [*t*(44) = 0.0365, *P* = 0.99]. These results demonstrate that α5-GABA_A_Rs on interneurons are essential for suppression of contextual fear conditioning by a sedative dose of etomidate.

To test whether α5-GABA_A_Rs on pyramidal neurons also contribute to etomidate's suppression of contextual fear conditioning, we similarly performed CPFE experiments in α5-pyr-KO mice and their p-WT littermates (p-WT_pyr_). Etomidate produced equal levels of sedation (day 1) in p-WT_pyr_ and α5-pyr-KO mice [Fig. 2C, *left*; drug × genotype, *F*(1, 61) = 0.19, *P* = 0.66]. Again, etomidate differentially suppressed freezing (day 3) in the two genotypes [Fig. 2C, *right*; drug × genotype, *F*(1, 61) = 14.17, *P* < 0.001], with significant suppression in p-WT_pyr_ mice [*t*(61) = 5.06, *P* < 0.0001] but not in α5-pyr-KO mice [*t*(61) = 0.233, *P* = 0.97]. This finding indicates that etomidate also engages pyramidal neuron α5-GABA_A_Rs to block memory, consistent with previous experiments linking these receptors to the control of spatial memory (48).

### Etomidate suppresses place cell formation via interneuronal α5-GABA_A_Rs

The hippocampus supports contextual memory by forming a cognitive map of the environment (49, 50). Spatially modulated firing of individual cells, referred to as “place cells,” contribute to that map (51, 52). Their spatial specificity can be stable for periods of days or even weeks in a given environment (53, 54), but they undergo “remapping” when the animal is placed in a new environment (55, 56). Although not every place cell is seen again upon reexposure to the same environment, when it is reactivated, its place field is retained (57). Therefore, as an ensemble, the formation and reactivation of place cells can serve as a “readout” of the encoded contextual memory, stable over a period of weeks (58).

**Fig. 3.**
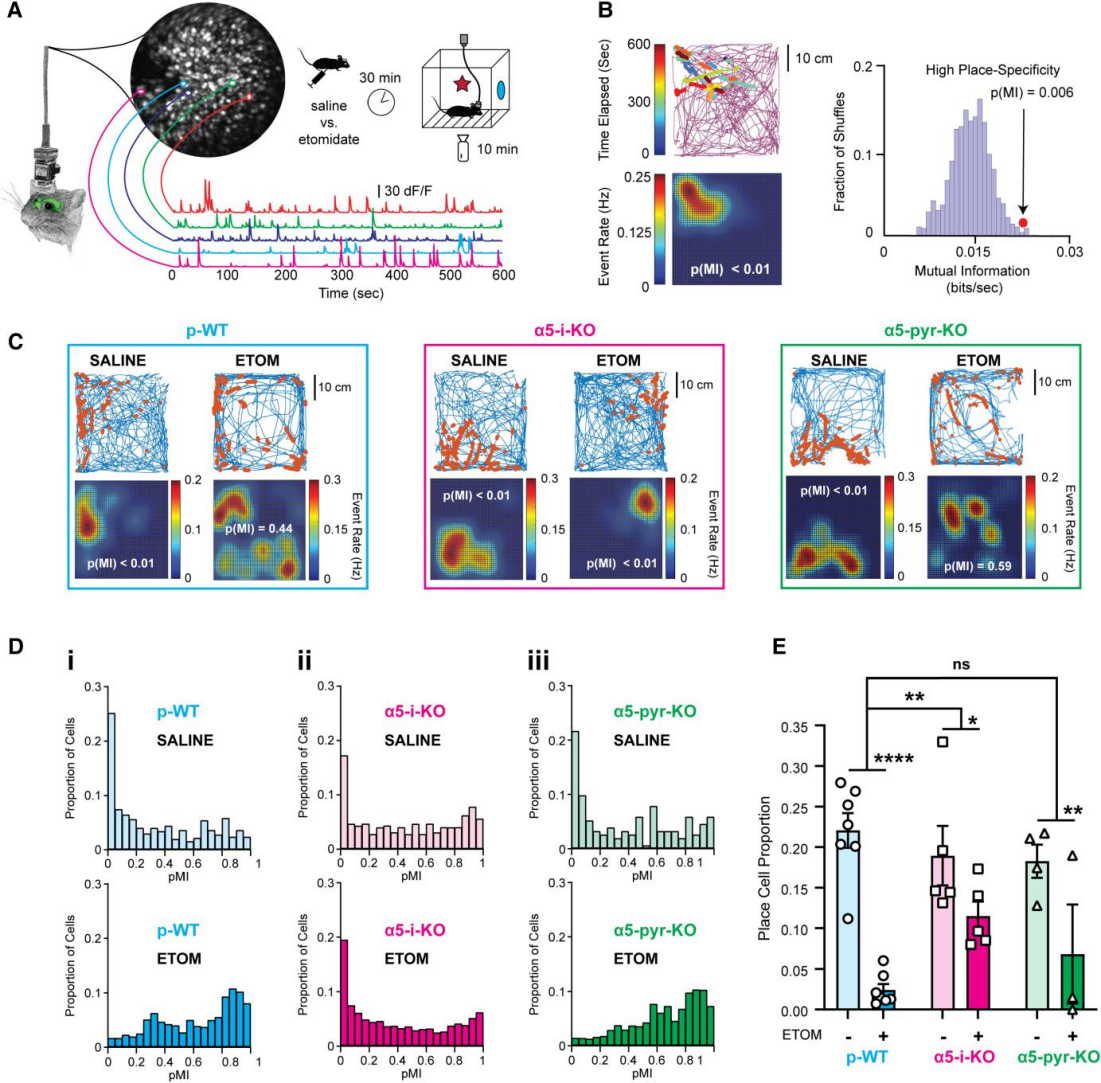
Suppression of place cell formation by etomidate is attenuated in α5-i-KO but not α5-pyr-KO mice. A) Hippocampal dorsal CA1 pyramidal neuron activity was captured using a head-mounted miniaturized endoscope to monitor AAV1-CaMKIIα-GCaMP6f fluorescence. Contours of five cells are overlayed on the maximum-projected image from a 10-min recording; their associated fluorescence traces are shown below. Upper right: experimental paradigm used to evaluate place cell formation. Ca^2+^ dynamics and arena location were captured simultaneously. B) Establishing the place-specific firing of a cell. Upper left: event dot map shows the location of the mouse when Ca^2+^ events were detected. The continuous line shows the track of the mouse in the experimental arena during the entire 10 min. Each string of dots represents a single Ca^2+^ event distributed along the rising phase of the Ca^2+^ transient, and the number of dots in each string reflects the event's adjusted amplitude. The string color designates the time at which the event occurred. Lower left: Gaussian-smoothed event rate map of the corresponding event dot map above. Warmer colors represent higher event rates. Right: mutual information (MI) between mouse position and firing rate for the cell shown on the left versus its time-shuffled null distribution. In this example, the probability of the observed MI (arrow and dots) falling within the null distribution was *P*(MI) = 0.006, which classifies the cell as a place cell. C) Examples of event dot maps and their derived event rate maps for representative cells after mice had been administered saline or etomidate (7 mg/kg). Lines show the track of the mouse and dots represent calcium events. Left: place-specific firing of a cell in a p-WT mouse under control conditions [*P*(MI) < 0.01] but not after etomidate [*P*(MI) = 0.44]. Middle: place-specific firing of a cell from an α5-i-KO mouse after saline [*P*(MI) < 0.01] and also after etomidate [*P*(MI) < 0.01]. Right: similar to p-WT, place-specific firing in a α5-pyr-KO mouse after saline [*P*(MI) < 0.01] but not after etomidate [*P*(MI) = 0.59]. D) *P*(MI) distributions of all cells from six representative sessions after saline or etomidate (7 mg/kg). Left (i): the left-skewed distribution of *P*(MI) (e.g. more place-specific cells) of a p-WT mouse under saline shifts to the right (e.g. cells losing place specificity) under etomidate. Middle (ii): the distribution of *P*(MI) retains its left-skewed shape despite of the presence of etomidate in an α5-i-KO mouse. Right (iii): similar to p-WT, the *P*(MI) distribution of an α5-pyr-KO mouse shifts to the right under etomidate. E) The effect of etomidate 7 mg/kg on place cell formation summarized for all three genotypes. Etomidate’s suppression of place cell formation was attenuated in α5-i-KO mice but not α5-pyr-KO mice. There was no influence of genotype on place cell proportions after saline [*F*(2, 12.45) = 0.31, *P* = 0.74] (*P < 0.05, **P < 0.01, ****P < 0.0001).

To test the influence of etomidate on this process, and whether it depends on α5-GABA_A_Rs on interneurons and/or pyramidal neurons, we used in vivo Ca^2+^ imaging in freely moving mice to assess the spatially modulated firing characteristics of dorsal CA1 pyramidal neurons in p-WT, α5-i-KO, and α5-pyr-KO mice as they explored novel contexts two to three times per week for up to 10 weeks (59). To capture Ca^2+^ signals, we injected virus carrying the genetically encoded calcium indicator GCaMP6f driven by the CaMKIIα promoter (AAV1-CaMKIIα-GCaMP6f) into the dorsal hippocampus of 16 mice (7 p-WT, 5 α5-i-KO, and 4 α5-pyr-KO). Two to three weeks later, a gradient refractive index (GRIN) lens and baseplate were attached to the skull, to which was affixed a miniature endoscope (nVoke2, Inscopix, Palo Alto, CA, USA). Beginning ∼4–6 weeks after virus injection, transient fluorescent signals were observed in up to ∼800 individual CA1 pyramidal neurons (Fig. 3A and Figure S3). Over the course of a 10-min period of free exploration in a novel environment, overall Ca^2+^ event rates averaged ∼0.1 s, similar to previous reports (57, 60).

To quantify place-specific firing of individual neurons, we simultaneously monitored the position of the mouse using video tracking software (Noldus EthoVision XT15) together with endoscopic fluorescent report of cellular activity (Inscopix Data Acquisition Software 2019). Figure 3B shows an example of one such experiment. The track of the mouse over the 10-min exploration is shown by the purple line, with dots superimposed over the rising phase of detected calcium events (Fig. 3B, *upper left*). This information was used to create a map of event rate as a function of position (Fig. 3B, *lower left*). To quantify the place specificity of Ca^2+^ dynamics of a specific cell, we calculated the mutual information (MI) between the cell's event map and mouse's spatial location map and compared it to a null distribution of MI values obtained from 1,000 time-shuffled event maps (Fig. 3B, *right* and Fig. S4A). Using the criterion that the probability of the observed MI value must fall within the top 5% of the null distribution (i.e. *P*(MI) < 0.05) for a cell to be considered a “place cell,” we found that in p-WT mice under control conditions, 22 ± 2% of all active cells (5 Ca^2+^ events over a 10-min recording) qualified as place cells, consistent with previous reports using this recording method (57).

≥A summary of the place cell proportions seen in all mice administered saline or 7 mg/kg etomidate is presented in Fig. 3E. On average, etomidate reduced the fraction of place cells from 22 ± 2% (saline) to 2 ± 1% in p-WT mice [*t*(46.23) = −8.87, *P* < 0.0001], whereas in α5-i-KO mice, the place cell fraction was reduced from 19 ± 5% (saline) to 12 ± 2% [*t*(25.99) = −2.56, *P* = 0.016], a significantly weaker effect [drug × genotype, *t*(103.69) = 2.74, *P* = 0.0071]. In α5-pyr-KO mice, etomidate reduced the place cell proportion from 18 ± 2% (saline) to 7 ± 5% [*t*(21.17) = −3.11, *P* = 0.0028], an effect that was not significantly different than p-WT [drug × genotype, *t*(114.23) = 1.065, *P* = 0.29].

To establish the dose dependence of place cell suppression, we carried out this same type of experiment using doses of 2, 4, 6, and 8 mg/kg etomidate. The findings mirrored those presented above, with strong dose-dependent suppression of place cell proportion in p-WT and α5-pyr-KO mice and resistance to this effect in α5-i-KO mice (Figure S7). The finding that place cell suppression depends on α5-GABA_A_Rs on interneurons mirrors our CPFE results (Fig. 2), supporting a causal link between etomidate's interference with place cell formation and hippocampus-dependent contextual learning. By contrast, the sensitivity of α5-pyr-KO mice to etomidate's suppression of place cells suggests that freezing in the CPFE experiments may have reflected a learning strategy that does not rely in the same way on spatially modulated firing.

### Etomidate suppresses spatial engrams via interneuronal α5-GABA_A_Rs

**Fig. 4.**
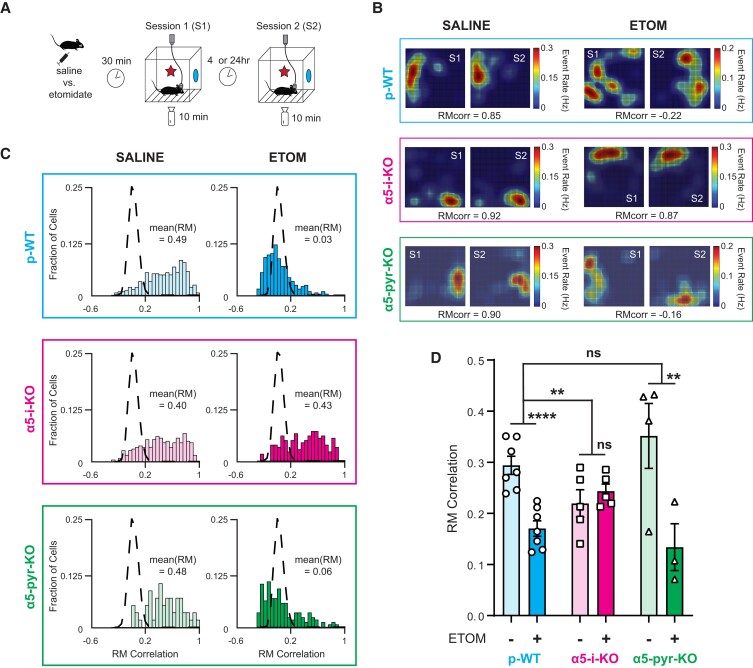
Etomidate suppresses hippocampal spatial engram stability in p-WT and α5-pyr-KO mice but not in α5-pyr-KO mice. A) Experimental paradigm used to assess spatial engram formation. The same mice used to assess place cell formation (Fig. 3) were reexposed to the same contexts in a second session. Saline or etomidate was administered only before the first session. B) Representative examples of event rate maps for three individual neurons from p-WT (top), α5-i-KO (middle), and α5-pyr-KO (bottom) mice that were administered saline (left) or 7 mg/kg etomidate (right). Their associated rate map (RM) correlation between S1 and S2 are shown below. Under control conditions (saline), cells retained their place specificity. After etomidate, cells in p-WT and α5-pyr-KO, but not α5-i-KO mice, underwent remapping. C) Distributions of RM correlations from six paired recording sessions in p-WT (top), α5-i-KO (middle), and α5-pyr-KO (bottom) mice that were administered saline (left) or 7 mg/kg etomidate (right). Left: under control conditions, distributions fell substantially to the right of the location-shuffled RM null distributions (dashed lines) in all three genotypes, revealing the presence of stable spatial engrams. Right: etomidate caused the distributions to be shifted toward the null distributions in p-WT and α5-pyr-KO mice but not in α5-i-KO mice. D) Summarized mean RM correlation for all three genotypes under saline and etomidate 7 mg/kg conditions. Etomidate strong impaired spatial engram stability in both p-WT and α5-pyr-KO mice but not in α5-i-KO mice (**P < 0.01, ****P < 0.0001).

Once formed, place cells can remain stable for days to weeks, displaying the same position-specific activity upon repeated exposure to the same environment (58), thereby forming a memory trace, or “spatial engram,” of the environment. Cells that do not meet the relatively strict criterion of *P*(MI) < 0.05 also carry some spatial information and contribute to the spatial engram (58, 61, 62). Therefore, to test the influence of etomidate on spatial engram stability, we measured the similarity between firing patterns during a mouse's initial exposure to a novel environment in “Session 1” (S1) versus its reexposure to the same environment 4 or 24 h later in “Session 2” (S2), using event RMs from all cells that were active during both sessions (Fig. 4A). We quantified the similarity of spatially modulated calcium dynamics in two ways: first, on a cell-by-cell basis, by computing the Pearson's correlation coefficient (PCC) between RMs formed by same cells in S1 and S2 (Fig. S4B); and second, on an ensemble basis, by computing the PCC between population vectors (PVs) of firing rates of all cells at each position in the arena (divided into 15 × 15 pixels) (Fig. S4C). Results for these two methods were essentially identical, so here we present RM_corr_ results; PV_corr_ results are presented in Fig. S8.

Representative examples of event RMs and their associated RM correlation values (RM_corr_) from paired sessions are shown in Fig. 4B. These cells exemplified the activity patterns we observed: under control conditions (saline) for all three genotypes, cells tended to fire in the same spatial locations when mice were returned to the arena (Fig. 4B, *left*). In p-WT and α5-pyr-KO mice given 7 mg/kg etomidate 30 min prior to S1, cells globally “remapped” to new locations, but α5-i-KO mice maintained consistent spatial activity patterns (Fig. 4B, *right*). This resistance of α5-i-KO mice to etomidate was evident in the distributions of RM_corr_ values of all cells seen in both sessions (Fig. 4C): RM_corr_ values fell largely outside the location-shuffled null distribution (dashed lines) when mice were administered saline, indicating that they had formed stable spatial maps under control conditions (Fig. 4C, *left*); in contrast, 7 mg/kg etomidate shifted RM_corr_ distributions toward null distribution in p-WT and α5-pyr-KO mice, but not in α5-i-KO mice (Fig. 4C, *right*).

Combined results from all such paired recording experiments in all genotypes, with saline or 7 mg/kg etomidate administered prior to S1, are presented in Fig. 4D. Etomidate significantly reduced RM_corr_ in p-WT [*t*(45.69) = −5.20, *P* < 0.0001] and α5-pyr-KO mice [*t*(26.04) = −2.98, *P* = 0.0062], but not in α5-i-KO mice [*t*(37.31) = 0.727, *P* = 0.472]. This drug effect was significantly different in α5-i-KO mice versus p-WT [drug × genotype; *t*(113.20) = 3.095, *P* = 0.0025] but not in α5-pyr-KO mice [*t*(108.08) = −0.628, *P* = 0.53]. Interestingly, the average RM_corr_ for α5-i-KO mice (0.22 ± 0.03) under saline control condition was significantly lower than that for p-WT mice [0.29 ± 0.02; *t*(59.19) = −2.26, *P* = 0.027], indicating that α5-GABA_A_Rs on interneurons play a physiological role in promoting spatial memory formation and/or stability. Similar experiments using etomidate doses of 2, 4, 6, and 8 mg/kg revealed dose-dependent reductions in RM_corr_ and PV_corr_ that were attenuated in α5-i-KO but not in α5-pyr-KO mice (Figures S7 and S8). These results show that in addition to interfering with place cell formation, etomidate suppresses spatial engram formation and/or stability through α5-GABA_A_Rs on interneurons but not pyramidal neurons.

The analysis described above relies on similarities between spatially modulated firing patterns to establish the presence of a “spatial engram.” However, the traditionally recognized engram is based not on spatially modulated firing but on overall firing rates during the entire period of exploration (learning), high enough to activate early immediate genes (IEGs—cFos, Egr1, ARC, and PNAS4) (63–65). To test whether a similar phenomenon might be present in the Ca^2+^ events that we measured here, we examined the effect of etomidate on the probability that a cell seen in S1 would also be active in S2 (cell recurring probability). We found no significant effects of etomidate in any of the three genotypes, though there was a trend toward lower cell recurring probability in p-WT and α5-pyr-KO mice but not in α5-i-KO mice (Figure S10). We conclude that IEG-defined engrams are distinct from spatial engrams based on overall Ca^2+^ event rates.

### Hippocampal spatial engrams are sensitive to local contextual cues and their representations of distal cues slowly evolve over weeks

**Fig. 5.**
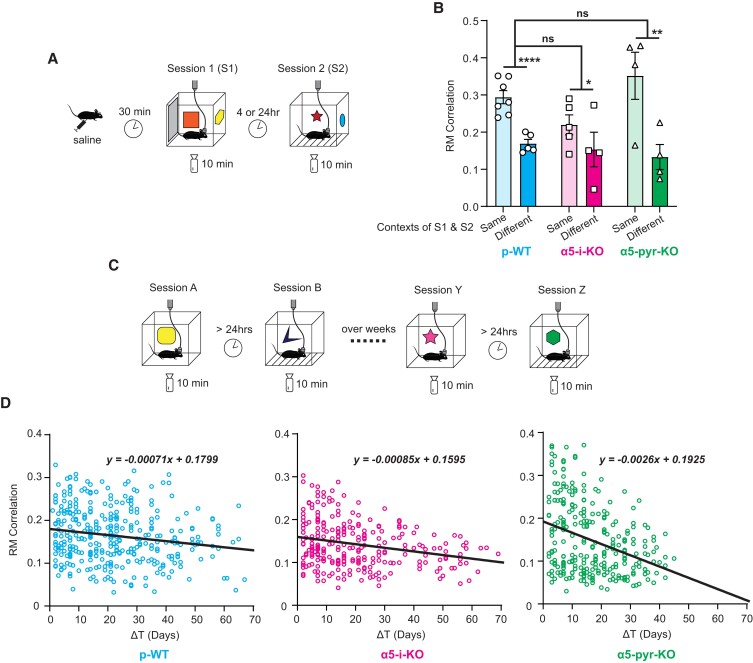
Hippocampal spatial engrams display context specificity. A) Experimental paradigm used to investigate context specificity of PV and RM correlations as contextual memory correlates. Mice were injected with saline 30 min prior to S1, and local contextual cues were changed before S2. B) Summarized results from different-context experiments. Changing contextual cues significantly (and similarly) reduced RM correlations for all three genotypes (**P < 0.01, ****P < 0.0001). C) Experimental paradigm used to investigate the “residual correlation” observed in different-context experiments in part A. Mice were exposed to novel contexts (different local cues) over weeks in the same general environment (identical distal cues). D) Residual RM correlation “drift” over weeks for all three genotypes with derived linear regression models. “Residual correlations” were higher when two sessions were conducted closer in time, and *y*-intercepts of the linear regression models closely match results obtained in different-context experiments in part A. α5-pyr-KO mice had significantly faster drift and significantly higher “residual correlations” at early time points (*y*-intercept).

To confirm that the RM and PV correlations reflect the formation of spatial engrams specific to the set of contextual cues presented during S1, we conducted an additional series of experiments using different contexts between S1 and S2 following saline administration (Fig. 5A). As shown in Fig. 5B, RM_corr_ values were indeed significantly lower in all genotypes when the contextual cues were changed between S1 and S2 [p-WT: *t*(32.97) = −4.46, *P* < 0.0001; α5-i-KO: *t*(21.75) = −2.37, *P* = 0.027; α5-pyr-KO: *t*(7.27) = −3.76, *P* = 0.0066]. There were no significant differences in this measure between genotypes [genotype × experimental condition; α5-i-KO: *t*(65.04) = 1.20, *P* = 0.23; α5-pyr-KO: *t*(56.82) = −1.52, *P* = 0.13]. Similar statistical conclusions were made with PV_corr_ (Figure S11). These findings support the use of RM and PV correlations as neuronal correlates of spatial memory.

Although both RM and PV correlations were reduced in different-context experiments compared to same-context experiments, they still were significantly higher than “0,” i.e. the location-shuffled null values (Fig. 4C, dashed lines). These “residual correlations” suggest that some component of the hippocampal activity pattern reflects a more general aspect of the environment (distal cues), such as the room in which experiments were conducted or the plexiglass arena surrounded by a blackout curtain into which the mice were placed, as opposed to the unique sensory cues within the arena that constituted the “context” (local cues) (66). Because we had always presented a new set of local cues for every S1 and S2 pair, we were able to conduct analyses analogous to the different-context experiments by treating each S2 as a unique recording session with novel local cues yet identical distal cues, thereby analyzing the “residual correlations” for up to 10 weeks (Fig. 5C). We observed that there was a slow “drift” of the “residual correlations” over weeks for all three genotypes, as shown by linear regression models with significantly nonzero slopes [p-WT: *F*(1, 361) = 11.36, *P* = 0.0008; α5-i-KO: *F*(1, 272) = 25.31, *P* < 0.0001; α5-pyr-KO: *F*(1, 275) = 36.91, *P* < 0.0001] (Fig. 5D). Moreover, “residual correlations” were greater when two sessions were close in time (e.g. 5 days), as opposed to farther in time (e.g. 60 days). The *y*-intercepts of these linear models represent predicted mean RM_corr_ of different-context experiments conducted close in time, and as expected, the *y*-intercepts closely matched those we obtained in different-context experiments. However, α5-pyr-KO mice had faster “drift” in “residual correlations” compared to p-WT and α5-i-KO mice (95% CI of linear regression model slopes: p-WT = −0.0011 to −0.00029; α5-i-KO = −0.0012 to −0.00051; α5-pyr-KO = −0.0035 to −0.0018). Again, PV correlations provided similar findings (Fig. S12). These findings lead us to speculate that spatial engrams are formed by a combination of experimentally altered local cues and unaltered distal cues (i.e. different levels in a hierarchical memory system (67)) and that the representation of the environment drifts over a time frame corresponding to slow components of system consolidation (68).

We conducted a final set of experiments analogous to the same-context (S1–S2 pair) experiments but using a separate group of four WT mice, where pairs of sessions were separated by either 4 or 24 h, to test whether spatial engrams have similar stability over 4 h versus 24 h (Figure S13). RM and PV correlations were essentially identical, indicating that the spatial engrams measured at 4 h provide a good measure of their strength at 24 h (and vice versa)—a more typical measure of long-term memory in behavioral studies, including those that we used for CPFE tests of memory (Fig. 2).

## Discussion

Results from both behavioral and Ca^2+^ imaging experiments demonstrated that α5-GABA_A_Rs on interneurons are essential targets for learning suppression by the archetypal GABAergic general anesthetic etomidate. The calcium imaging results further showed that in addition to this pharmacological role, interneuronal α5-GABA_A_Rs play a physiological role in promoting spatial learning. Ca^2+^ imaging experiments provided additional mechanistic insights by showing that etomidate suppresses the initial development of place cells and interferes with the formation and/or stability of a spatial engram encoded by a combination of place cells and nonplace cells. An essential role for interneuronal α5-GABA_A_Rs in etomidate-induced memory suppression challenges the longstanding and widely accepted model centered on tonic and slow phasic inhibition on pyramidal neurons (29–31, 34, 69, 70).

The role of α5-GABA_A_Rs on pyramidal neurons is less clear. Insofar as spatial engrams serve as neural correlates of hippocampal memories, our results from α5-pyr-KO mice, in which etomidate was still able to suppress engrams (Fig. 4), indicate that modulation of interneuronal α5-GABA_A_Rs alone is sufficient. However, selective elimination from pyramidal neurons did prevent etomidate from suppressing freezing in the CPFE paradigm (Fig. 2), and suppression of place cell formation was only partially attenuated in α5-i-KO mice (Fig. 3 and Figure S7). Therefore, etomidate's full effect on memory may derive from partial contributions of both interneuronal and pyramidal neuron α5-GABA_A_Rs.

If etomidate prevented α5-pyr-KO mice from forming a stable hippocampal contextual representation (Fig. 4), how then were they still able to associate the arena with the footshock (Fig. 2)? A likely explanation is that mice usually rely upon hippocampus to overcome the immediate shock deficit in CPFE (46, 47), but when the hippocampus is damaged or impaired prior to training, mice can form context-shock associations using other brain regions and learning strategies (71–73). Extra-hippocampal regions that mediate these alternate learning strategies include medial prefrontal cortex (74), retrosplenial cortex (75–77), and dorsolateral striatum (78). It is also possible that the neural correlates that we focused on here do not completely capture the behaviorally relevant contributions of hippocampal activity to shock-elicited freezing. Indeed, others have reported that neural correlates in some learning tasks correspond better than others to behavioral readouts, such as discrimination tasks that depend on pattern separation in DG, but for which behaviors are more consistently aligned with CA1 firing (79). Also, hippocampal spatial mapping occurs differently when mice navigate in a multisensory environment compared to one that engages only a single sensory modality (80); our use of a multisensory-rich cue set in Ca^2+^ experiments compared to only visual cues in CPFE experiments might therefore have influenced our findings.

The methods we used—repeatedly exposing mice to novel sets of contextual cues several times per week for up to 12 weeks—provided robust cellular correlates of spatial memory, and they allowed us to develop full dose–response relationships from groups of only four to six mice of each genotype. Not only should this approach be applicable to a wide range of questions, but it also has the added benefits of efficient resource utilization and minimization of animal welfare concerns by reducing the total number of animals used.

Here, we have used the term “spatial engram” to refer to the correspondence between the recurrence of spatially modulated neuronal activity patterns (Fig. 4) and the formation or suppression of hippocampus-dependent contextual memories (Fig. 2). In this regard, spatial engrams incorporate characteristics of both place fields as cognitive maps (49, 81) and engrams as revealed and captured by the activation of learning-related IEGs and replayed through optogenetic activators (63–65). However, spatial engrams differ from place cell maps and IEG-defined engrams (a collection of engram cells) in several important ways: (i) they include both place cells and nonplace cells; (ii) they presumably include both engram cells and nonengram cells (61, 82); and (iii) they represent ensemble activity of a large proportion of the population rather than a restricted subset of highly active cells. These spatially modulated firing patterns, which other investigators who have used the same recording methods have also observed (57, 58, 66, 83), embody many of the characteristics expected of neuronal correlates of memory. These include the ability to retain stable representations of an environment over extended periods of time (57, 66), the ability to discriminate between arenas with different cues but at the same time to generalize between arenas in the same general environment (66) (Fig. 5), and fluctuating activity patterns that create unique episodic time stamps (84). Here, we add to this collection of attributes the finding that drugs that prevent learning also directly interfere with the development of stable neuronal ensembles—thus extending it from a set of correlations to include an interventional test. Admittedly, these findings do not yet match the extensive evidence developed over the past decade indicating that IEG-captured engram cells represent the physical substrate of memories themselves (64). To develop a comparable level of evidence would require reinstating the activity of the entire ensemble in a manner that drives memory-guided behavior, but such a method has not yet been developed. Nevertheless, it is likely that spatial engrams do include IEG-captured engram cells; ecphory might therefore occur, but this point is speculative. Furthermore, just as a set of bona fide engram cells observed in the hippocampus or elsewhere in the brain does not constitute an entire memory trace but is just one portion of an engram complex (85), the spatial engrams that we describe here are likely one component of a memory trace that extends across multiple structures (86), to capture the memory of the arena, the general environment, and the context within which the experiments take place.

Which types of interneurons might be responsible for the present findings? It is counterintuitive that suppressing interneuron activity through positive modulation of their α5-GABA_A_Rs would impair memory—at least if one considers inhibitory interneurons only in terms of controlling the depolarization of pyramidal neurons. Reducing this constraining influence would instead lead to net disinhibition, enhancing the ability of pyramidal neurons to generate dendritic bursts, become place cells, and establish networks that store memories (34, 87, 88). However, interneurons also target other interneurons, creating positive feedback loops that can support the induction of LTP and associative learning and memory (89, 90). Interrupting these disinhibitory loops might then be the key. In this regard, interneurons that express vasoactive intestinal peptide (VIP+ INs) are obvious candidates since they preferentially target other interneurons. However, VIP+ INs were reported to not express α5-GABA_A_Rs in human or mouse prefrontal cortex (11), and an examination of the distribution of Gabra5 mRNA in hippocampal CA1 interneurons using a published database (91) confirms that this is the case in hippocampus as well, as Gabra5 levels are lowest in Vip+ of all interneuron classes (Fig. S14). At the other end of the Gabra5-expression spectrum are Cck+ basket cells—their Gabra5 expression levels are the highest of all (Fig. S14), they are densely expressed in dorsal CA1 (92), and their selective activation has been shown to enhance memory (93). Therefore, depression of Cck+ basket cells via α5-GABA_A_Rs is one possible mechanism. Neurogliaform and ivy cells also express Gabra5, at intermediate levels (Fig. S14), and they also receive GABA_A_R-mediated slow IPSCs (94), which on pyramidal neurons are mediated by α5-GABA_A_Rs (34, 69, 95). In addition, they have high-density release sites, and they inhibit other interneurons as well as pyramidal cells via firing patterns that are time-locked to the theta oscillation (94, 96). Therefore, these cells may also be involved. Finally, Sst+ INs, which receive α5-GABA_A_R, _slow_ IPSCs (97) and play essential roles in the formation and control of contextual fear conditioning (98, 99), are also good candidates for the control of learning and memory through interneuronal α5-GABA_A_Rs.

A role for α5-GABA_A_Rs located on interneurons in controlling hippocampus-dependent memory may have wide ramifications. Changes in α5-GABA_A_R expression or activation have been proposed to contribute to memory deficits in Alzheimer's disease, schizophrenia, autism, and aging and in the early postoperative period (24, 100–104). Even if they are not the pathological basis per se, modulation of α5-GABA_A_Rs has also been found effective in preclinical models of depression and Down syndrome (21–23). It should be noted that our findings also support important roles for α5-GABA_A_Rs on pyramidal neurons; indeed, there is evidence that pyramidal neuron α5-GABA_A_Rs specifically regulate certain memory domains (48). However, most studies focused on cognition have assumed that effects come from direct modulation of pyramidal neurons; the idea that they might derive instead (or in addition) from modulation of interneurons opens interesting possibilities. For example, modulation of α5-GABA_A_Rs on different cell types might help explain why both α5-PAMs and α5-NAMs show efficacy in treatment of animal models of depression (20, 21, 23). The recognition that α5-GABA_A_Rs on interneurons are essential components of the hippocampal memory machinery, together with an emerging understanding of the functional diversity of interneurons, should present new opportunities for discovery and development of targeted therapeutics.

## Materials and methods

### Animals

All experiments were conducted under the oversight and with the approval of the Institutional Animal Care and Use Committee (IACUC) of the University of Wisconsin-Madison. Contextual fear conditioning experiments (CPFE) were conducted on a total of 74 mice from the GAD2-Cre (GAD2^tm2(Cre)Zjh^) × α5-floxed (Gabra5^tm2.1Uru^) line and 90 mice from the CaMKIIα-Cre (Tg(Camk2a-Cre)T29-1Stl) × α5-floxed (Gabra5^tm2.1Uru^) line. Ca^2+^ imaging experiments (GCaMP6f) were conducted on seven p-WT mice (α5-floxed, Cre−), five α5-i-KO mice, four α5-pyr-KO mice, and four wild-type C57BL6/J mice. Mice of both sexes were used for CPFE experiments (85 females, 79 males, ages 60–150 days). Only male mice were used for GCaMP6f imaging (ages 78–131 days at the time of imaging) because their larger size facilitated lens and baseplate implantation.

All mice were generated and maintained on the C57BL/6J background. p-WT and their interneuron-specific Gabra5 knockout littermates (α5-i-KO and α5-pyr-KO) were created in a three-step process. F0 mice were homozygous α5-floxed (α5:fl/fl) kindly provided by the University of Zurich (105), homozygous GAD2-IRES-Cre (JAX strain # 010802) (106), hemizygous Viaat-Cre (JAX strain # 017535) (107), or homozygous CaMKIIα-Cre (JAX strain # 005359) (108) mice obtained from the Jackson Laboratory (Bar Harbor, Maine, USA). As expected, F1 offspring were all heterozygous for the α5-floxed gene (α5:fl/−) and the GAD2-Cre or CaMKIIα-Cre gene, and approximately one-half were also hemizygous for the or Viaat-Cre gene. F2 mice were created by crossing a F1 α5:fl/−;Cre+ mouse and a F1 α5:fl/−;Cre− mouse. F3 mice were created by crossing F2 mice that were homozygous for α5-floxed gene (α5:fl/fl;Cre−) with F2 mice also homozygous for α5-floxed gene plus hemizygous for GAD2-Cre, Viaat-Cre, or CaMKIIα-Cre genes (α5:fl/fl;Cre+). F3 mice consisting of (α5:fl/fl;Cre+) and (α5:fl/fl;Cre−) were used as breeding pairs. Experimental mice came from F3 breeding pairs and subsequent generations produced by the same strategy (α5:fl/fl;Cre+) × (α5:fl/fl;Cre−).

We monitored for germ-line recombination by testing for the presence of Cre recombinase plus the α5-floxed allele, the α5-KO allele, and the α5-WT allele. Any mice that were negative for Cre recombinase but positive for the α5-KO allele were considered to have undergone germ-line recombination. We observed a high rate of germ-line recombination in the Viaat-Cre × α5-floxed line, whether Cre was carried by the male or female (∼25% of offspring), but never any germ-line recombination in the GAD2-Cre × α5-floxed or CaMKIIα-Cre × α5-floxed lines, whether Cre was carried by the male or female. Therefore, all experiments were conducted using only mice from the GAD2 and CaMKIIα lines.

### Genotyping

All experimental mice were genotyped from tail samples either in-house using traditional, gel-based PCR methods, or sent to Transnetyx (Cordova, TN, USA), which uses a TaqMan-based assay for real-time PCR data. For in-house PCR, primers purchased from IDT (Integrated DNA Technologies, Coralville, IA, USA) were as follows (forward, reverse):

Gabra5: TGATGGCACACTTCTCTACACC, CTTTGAAAGCATTTCCCGAAGC; GAD2-Cre: CTAGGCCACAGAATTGAAAGATCT, GTAGGTGGAAATTCTAGCATCATCC; and Viaat-Cre: GCGGTCTGGCAGTAAAAACTATC, GTGAAACAGCATTGCTGTCACTT.

### Ex vivo microscopy

To image fluorescent markers td-Tomato and GFP, coronal slices (100- to 400-um thickness) were cut from three mice and imaged live in cold, oxygenated ACSF. Hippocampal subfields were imaged on an Olympus FluoView FV1000 upright confocal microscope with a water immersion objective (either a LUMPlanFl/IR 40× N.A. = 0.8, or a XLUMPLFL 20× N.A. = 1.00). Z-series images (at least 40–50 μm in Z) were acquired with 488- and 561-nm laser lines (sequential excitation). Several dozen fluorophore-expressing cells were observed in each volume of interest. From these Z-series, the cell bodies of fluorophore-expressing cells (either td-Tomato or GFP) were segmented, and average fluorescence (a.u.) in each color was quantified.

### Tissue sectioning for IHC

Mice were anesthetized by isoflurane inhalation and transcardially perfused with 4% paraformaldehyde (PFA)/phosphate-buffered saline (PBS). Post-fixation, brains were incubated in 4% PFA/PBS for 4 h followed by an overnight incubation in 30% sucrose/PBS at 4°C. Prior to sectioning, brains were incubated in 4.5 pH Na-Citrate buffer overnight and then underwent microwave heating (900 W in 80 mL freshly prepared 6 pH Na-Citrate buffer for 90 s). The brains were then coronally sectioned at 30 μm with a vibratome (Leica VT1000s**)**.

### Immunohistochemistry

Slices were washed two times in PBS for 10 min and then incubated in primary antibody solution [0.2% Triton X-100, 10% normal goat serum (NGS; Thermo Fisher Scientific Cat# 50197Z), rabbit polyclonal anti-GABRA5 (1:200, Origene Cat# TA338505), and guinea pig polyclonal anti-GAD2 (1:200, Synaptic Systems Cat# 198 104) overnight at 4°C in a moist chamber with constant agitation (100 rpm)]. Slices were then washed three times in PBS for 15 min followed by a 45-min incubation in the secondary antibody solution (2% NGS, 1:200 goat anti-rabbit IgG (H + L) Alexa Fluor 568 Thermo Fisher Scientific Cat# A-11036 and 1:200 goat anti-guinea pig IgG (H + L) Alexa Fluor 647 Thermo Fisher Scientific Cat# A-21450) at room temperature (RT) with continuous agitation. Slices were then washed three times for 15 min for a final time before mounting on slides with a DAPI mounting medium (Abcam Cat# ab104139). Slides were imaged using a Nikon A1R HD upright multiphoton/confocal microscope.

### α5-GABA_A_R expression quantification

IHC images were taken from all hippocampal CA1 layers (*stratum oriens*, *stratum pyramidale*, *stratum radiatum*, *and stratum lacunosum-moleculare*) and loaded into Fiji v2.7.0 as Z-stacks consisting of three channels (DAPI, GAD2, and α5). For each Z-stack, the following analysis was applied: (i) the three channels were split into independent Z-stacks; (ii) each Z-stack was contrast-enhanced (% saturated pixels = 0.2%) and smoothed with a median filter (radius = 1 pixel) for better visualization and easier thresholding; (iii) cell-shaped GAD2+ neurons (candidate interneurons) were individually outlined as regions of interest (ROIs); (iv) ROIs were applied to the DAPI channel to confirm that each ROI contains a stained nucleus; (v) the “otsu threshold” function based on the stack histogram was applied to the α5 channel Z-stack to reduce background noise; (vi) the thresholded α5 channel Z-stack was “Z-projected” by maximum intensity; (vii) within each ROI identified and confirmed by steps (iii) and (iv), the number of punctate α5 particles in the maximum-projected image of the α5 channel was summarized in a metadata structure; and (viii) statistical analyses were carried out using GraphPad Prism v 9.3.1.

### Western blot analysis

A 1:2 dilution of supernatant of the anti-α5-GABA_A_R NeuroMab clone N415/24 was used. There were three animals per genotype and gender. Single animals from each group were run per gel (3 gels total). Expression levels were quantified by densitometry and normalized to β**-**actin (42 kDa). The means from the three WT males or three WT females, corrected for β-actin levels, were set to 1.0.

### Behavioral experiments

Behavioral studies were carried out at the Waisman Center Rodent Models Core facility at the University of Wisconsin-Madison. Mice were transferred from the primary animal care unit in which they were bred and raised to the Waisman Center animal care unit at least 1 week prior to initiating behavioral experiments. Studies of contextual fear conditioning were carried out first, followed by elevated plus maze, and then thermal sensitivity, with 3–4 days between experiments.

### Context preexposure facilitation effect

We used a preexposure-dependent contextual fear conditioning paradigm adapted from Cushman et al. (109). This paradigm, which is often referred to in the literature as the CPFE paradigm (42–44) takes advantage of the so-called immediate shock deficit (41, 45), wherein animals that are shocked immediately (within several seconds) upon entry into a novel environment do not freeze on subsequent reexposure, whereas mice that had been exposed on a prior day do exhibit a freezing response. The proposed explanation is that preexposed mice establish a hippocampus-dependent representation of the environment (i.e. contextual memory), which takes several minutes, and this preformed memory can be recalled rapidly on reexposure, for subsequent association with the aversive stimulus (46, 47). This paradigm was chosen because etomidate, as an anesthetic, might suppress freezing to context during a standard contextual conditioning paradigm, not by preventing the formation of a contextual memory (a hippocampus-dependent process), but rather by reducing the aversive nature of the shock, or some other aspect of its ability to support aversive conditioning. By administering etomidate during the context exposure phase, separated by 1 day from the shock exposure phase, that interpretive ambiguity is removed.

The CPFE experiments took place over 2 weeks. During the first week, the mice were habituated and handled in the behavioral testing room for 10 min a day. During the second week, the mice underwent three experimental phases—context preexposure, conditioning, and recall—on 3 consecutive days. Mice were brought into the behavioral testing room 30 min prior to the testing procedure. On day 1 (*context preexposure*), mice were injected with either saline or etomidate (7 mg/kg IP), placed back in their home cage for 30 min, and then placed into the test chamber for 10 min. The test chamber was 20 cm × 20 cm × 30 cm high, constructed of clear acrylic with checkered patterned paper covering three of the four walls and a shock grid floor consisting of stainless-steel bars 2 cm apart, diameter 2 mm. On day 2 (*conditioning*), mice were placed into the same test chamber, and after 15 s, they were administered a single footshock (2 s, 1 mA). The mice remained in the test chamber for an additional 30 s (47 s of total time) and they were then returned to their home cage. On day 3 (*recall*), mice were placed in the test chamber for 8 min. The amount of time they spent moving was recorded using FreezeFrame software. The percentage of time they were “immobile” (movement below a predefined threshold level) on day 1 served as a measure of drug-induced sedation; the percentage of time that they were immobile during the first 3 min of the drug-free test on experimental day 3 (“freezing behavior”) served as a quantitative measure of fear memory.

No significant differences between sexes were seen for either day 1 (sedation) or day 3 (freezing) for any groups (matching genotype and drug condition, unpaired *t*-test without correction for multiple comparisons), or in interaction between genotype (p-WT vs. KO) and drug condition (saline vs. ETOM) for either α5-i-KO or α5-pyr-KO lines (two-way ANOVA), so data from males and females were combined.

### Elevated plus maze test

Mice were placed individually in a plus-shaped maze composed of two “open” arms without walls (30 cm L × 5 cm W) and two closed arms (30 cm L × 5 cm W) enclosed by walls (10 cm H) arranged around a center zone (5 cm L × 5 cm W). Over the course of 5 min, the amount of time they spent on the arms and the number of entries to each arm were manually recorded by an experimenter blind to the genotype of the mice. The percent time the animal spent on the open arms and the number of entries to each arm served as a measure of anxiety-like behavior, with more entries and more time spent on the open arms indicating less anxiety.

### Hotplate test

An electronically controlled hotplate (30 cm L × 30 cm W) heated to 55°C was used to measure sensitivity to a noxious thermal stimulus. The hot plate was turned on 30 min prior to testing to ensure that the desired temperature was reached. Mice were then placed individually on the hot plate and the latency to elicit a nocifensive behavior (e.g. hind paw withdrawal or licking) was manually recorded by the experimenter blinded to the genotype of the mice.

### Ca^2+^ imaging experiments

Twenty male mice (7 p-WT, 5 α5-i-KO, 4 α5-pyr-KO, and 4 C57BL/6J) underwent two stereotaxic surgeries separated by 2–3 weeks. Mice were anesthetized using isoflurane. Postoperatively, they received subcutaneous carprofen 5 mg/kg for pain control.

For the first surgery, a virus carrying the genetically encoded calcium indicator GCaMP6f driven by the CaMKIIα promoter (Inscopix Ready-to-Image AAV1-CaMKIIα-GCaMP6f) was injected into the dorsal hippocampus. For the second surgery, 2–3 weeks later, the cortex and corpus callosum overlaying the hippocampus were aspirated using a 30-g blunt needle, and a baseplate with integrated GRIN lens was affixed to the skull. Two to three weeks later, a miniature epifluorescence microscope (Inscopix nVoke) was affixed to the baseplate, and the mouse was placed in the behavioral arena (a 40 cm × 40 cm × 30 cm tall acrylic enclosure) surrounded by blackout curtain. Settings for illumination, gain, and focus were established, and if there were >60 cells with at least five Ca^2+^ transients over a 10-min recording session, the mouse was used for Ca^2+^ imaging experiments.

On each experimental day, the nVoke camera was affixed to the baseplate, and the mouse was placed in a behavioral arena that contained visual, olfactory, and tactile cues, with a low level of illumination to encourage exploration. Paired experimental sessions, with mice exposed to a novel set of cues for the first session then the same set of cues for the second session, took place two to three times per week, with 4 or 24 h between sessions, and at least 1 day between successive pairs of sessions. Ca^2+^ activity was recorded and processed using Inscopix Data Processing Software v1.8.0 (IDPS v1.8.0), and mouse position within the arena was tracked using an IR-sensitive camera and Noldus Ethovision XT software.

Further analysis of place cells and spatial engrams took place using a combination of a MATLAB API package embedded within IDPS and custom-written MATLAB functions. Assessment of cellular activity and longitudinal registration of cell maps utilized the constrained nonnegative matrix factorization for microendoscopic data (CNMF-E) analysis, with analysis parameters optimized according to published methods (110) and visual inspections. Noisy calcium traces of detected cells were deconvolved using the “online active set method to infer spikes” (OASIS) method (111). For place cell and spatial engram analysis, we used custom-written MATLAB functions to merge behavioral tracking and calcium event data (Figure S4). For each cell, an amplitude-weighted *calcium event map* was created by duplicating calcium events according to their amplitudes (∼1–8 MAD) and distributing them along the track of the mouse, at intervals of 40 ms, over the rising phase of the GCaMP6f fluorescence signal, then a *calcium event RM* (events/s; 15 × 15 matrix) was created by dividing the calcium event map by the occupancy-time map, pixel by pixel. The MI between the calcium event RM and occupancy-time map for each cell was then calculated and compared to a null distribution of time-shuffled MI values to calculate *P*(MI) values for each cell. Spatial engrams based on a cell-by-cell basis (RM correlation) or on a location-by-location basis (PV correlation) employed PCC.

Please see online supplementary materials for additional detailed experimental and analytic methods.

### Data presentation and statistical analysis

Statistical analyses of α5-GABA_A_R expression in IHC images were done using two-tailed Mann–Whitney *U* test (GraphPad Prism v 9.3.1). Statistical comparisons for behavioral studies (CPFE, elevated plus maze, and hot plate) were made using two-way ANOVA followed by Šídák's multiple comparisons test, where interaction factors (e.g. drug and genotype) are reported as “A × B” (GraphPad Prism v 9.3.1). Comparisons for Ca^2+^ imaging data were made by fitting linear mixed effects models using the “lmer” package, with statistical significance derived from Kenward–Roger type III and likelihood ratio tests (112), implemented in RStudio, 2021.09.0 Build 351. To evaluate place cell proportions in single sessions, we used a model with drug (saline or etomidate dose) and genotypes as fixed effects, and mouse ID and experimental date as random effects. To evaluate RM_corr_ and PV_corr_ from pairs of sessions, we used a model with drug and genotypes as fixed effects, and cell ID (RM_corr_ only), mouse ID, and date as random effects.

## Supplementary Material

pgad065_Supplementary_Data

## Data Availability

All materials used in this study will be made available upon reasonable request from the lead contact. The data and analysis code generated in this study are available in a persistent repository (113).
