# Supplementary material for: Control of contextual memory through interneuronal α5-GABAA receptors
Source: PNAS Nexus. 2023 Apr 11;2(4):pgad065. doi: 10.1093/pnasnexus/pgad065 (PMC10089065; doi:10.1093/pnasnexus/pgad065)
Supplement: pgad065_Supplementary_Data [file pgad065_supplementary_data.pdf]

Zhu et al. (PNAS Nexus MS# PNASNEXUS-2022-01183-T)

Supplementary information

Supplementary Methods – Ca^{2+} imaging

Each mouse underwent two stereotaxic surgeries to prepare for measurement of dorsal hippocampal pyramidal neuron activity by Ca^{2+} imaging. For both procedures, mice were anesthetized with isoflurane ~2% adjusted to maintain immobility and spontaneous ventilation and warmed by block heater to maintain normothermia. Postoperatively mice received a subcutaneous injection of carprofen 5 mg/kg.

For the first surgery, a small craniotomy window was made over the right dorsal hippocampus (from bregma: AP = -2.0, ML = -1.6) and 500 nL of virus carrying the genetically encoded calcium indicator GCaMP6f driven by the CaMKIIα promoter (Inscopix Ready-to-Image AAV1-CaMKIIα-GCaMP6f) was injected at DV = -1.6 at a rate of 80 nL/min using a NanoFil syringe with a 35 g needle driven by a UMP3 microsyringe pump and SMARTouch controller (WPI). The needle was kept in place for 2 min, then retracted 200 um, where it remained for an additional 8 minutes before removing slowly. The skin was sutured closed, and the mouse was observed during recovery from surgery and anesthesia then returned to its home cage. For the second surgery, which took place 2-3 weeks later, a larger craniotomy was made at AP = -2.2, ML = -2.1, and the cortex corpus callosum overlaying the hippocampus was aspirated using a 30g blunt needle and cold saline irrigation, taking care not to aspirate the alveus. Once bleeding was controlled with Gelfoam and cold saline irrigation, an integrated GRIN lens (1 mm diameter x 4 mm length) and baseplate were inserted slowly at a 9-degree angle to the midline, until the center of the surface of the lens rested DV = -1.2. The baseplate was cemented in place using Metabond, the surrounding skin was sutured closed, and the mouse was allowed to recover from surgery and anesthesia, then returned to its home cage.

Two to three weeks later, a miniature epifluorescence microscope (Inscopix nVoke) was affixed to the baseplate, and the mouse was placed in the behavioral arena (a 40 cm x 40 cm x 30 cm tall acrylic enclosure) surrounded by blackout curtain, with exam table paper placed on the floor of the arena and allowed to explore freely for 10 minutes. A commutator (Inscopix) was used to prevent tangling of the wire attached to the microscope. During this initial ‘screening session’, the focal plane, illumination, and gain were adjusted to identify the optimal settings to observe cellular activity. If the number of cells with sufficient activity levels was too low (fewer than 60 cells with >5 Ca^{2+} events) the mouse was returned to its home cage and tested one week later. Once sufficient activity was observed, which typically occurred 5-6 weeks following the initial surgery with virus injection, the same settings were used for all subsequent recording sessions, except that the illumination was occasionally adjusted down or up to maintain appropriate fluorescent signal levels.

On each experimental day, two recording sessions separated by either 4 or 24 hours (Session 1 and 2, 10 min each) took place in the same behavioral arena used for the initial screening sessions. For Session 1, the arena contained a unique set of visual, olfactory, and tactile cues forming a ‘novel context’. Visual cues consisted of 8” x 11” sheets of paper mounted onto the outsides of all four walls, with different solid colors, shapes (stars, circles, squares, triangles) and patterns (stripes, swirls, lines) on the four sheets. Olfactory cues consisted of 1 μL of odorant (benzaldehyde, hexanal, alpha-pinene, heptaldehyde, eugenol, or eucalyptol) on filter paper placed within a 35 mm covered culture dish and set in one corner of the arena. Tactile cues consisted of ¼” thick acrylic squares covering one quarter of the arena floor, or various sizes of shallow glass petri dish lids placed either open side up or down, in addition to a sheet of exam table paper covering the floor of the entire arena. Prior to Session 1, a mouse received either saline (control) or etomidate (2-8 mg/kg) by intraperitoneal injection, carried out in a separate room. Thirty minutes after the injection the mouse was brought into the recording room, the miniature microscope was attached to the baseplate, the mouse was placed in the arena, and the light in the arena was turned down to ~2 lux to encourage exploration. A bank of infrared lights above the mouse (12 lux) was used to illuminate the arena so that the mouse could be tracked using an IR-sensitive camera (Basler aca1300-60 gm) mounted below the arena (Fig. 3A and 4A). The video camera and epifluorescent microscopy acquisition were synchronized using a hardware trigger controlled by Ethovision software (Noldus Information Technology). At the end of the 10-min recording session, the miniature microscope was detached, and the mouse was returned to its home cage in a separate room. For Session 2, the mouse was brought back into the recording room, the miniature microscope was mounted to the baseplate, and the mouse was placed back into the arena containing the same set of sensory cues used for Session 1.

Experiments using a novel set of cues for Session 1 and unchanged cues for Session 2 (same-context experiments) took place 2-3 times per week, with at least one day between two consecutive pairs of recording

sessions. For different-contexts experiments, altered sets of sensory cues were used for Session 1 vs. Session 2. For each mouse, experiments continued for up to 10 weeks (16 weeks post-virus injection), at which time the number of cells with sufficient activity was typically declining.

Behavioral tracking recordings were analyzed using Noldus EthoVision XT15 and exported as csv files. These csv files summarized the mice's XY-location (body center) as a function of time. All behavioral tracking csv files were organized into a specific data structure with experimental time, animal ID, genotype, and related experimental conditions. The in-vivo calcium imaging recordings were also organized into a matching data structure. An excel metadata sheet for all animals and all experiments allows for programmatic reference (MATLAB 2021a).

Initial processing of calcium imaging recordings. We utilized the MATLAB API package embedded within Inscopix Data Processing Software v1.8.0 (IDPS v1.8.0) for this initial stage of analysis. To begin, each raw recording was 'preprocessed' to remove artifacts, then both spatially and temporally downsampled by a factor of two to improve processing speed without compromising the resolution of cellular calcium dynamics. These recordings were then spatially filtered (parameters: low cutoff = 0.005 $pixel^{-1}$; high cutoff = 0.5 $pixel^{-1}$) to remove low and high spatial frequency content for better contrast and smoother frames, and frames were motion-corrected based on an arbitrarily chosen region of interest (ROI). To improve visualization of calcium events and prepare for the second-stage analysis by CNMF-E algorithm, a ΔF/F transformation was applied and a maximum projected image of the entire field of view was acquired.

Extraction of cellular activities and longitudinal registration of cell maps. The constrained nonnegative matrix factorization for microendoscopic data (CNMF-E) analysis was applied to the recordings after the initial processing stage, with analysis parameters optimized according to published methods (1) and visual inspections. Noisy calcium traces of detected cells were deconvolved using the 'online active set method to infer spikes' (OASIS) method (2). Once deconvolved, calcium events were inferred from calcium traces based on the biokinetics of GCaMP6f, using a threshold of 4 median absolute deviations (MAD) (3). Several analyzed cellsets were randomly chosen to verify that inferred spikes accurately reflected calcium signals from detected cells, based on visual inspection. To exclude non-cellular activities picked up by the CNMF-E algorithm, we required that detected cells must have exactly one spatial component, appropriate size, and more than 5 detected calcium events over a 10-minute recording session. Finally, cellsets of all recordings obtained from an individual mouse were longitudinally registered, so that the activity of each unique cell could be traced between and across recording sessions (4). These data were then exported as csv files that included global cell IDs, the timestamps (beginning of rising phase) of inferred calcium events, and the fluorescence amplitude of calcium events in MAD.

Place cell and spatial engram analysis. For the second stage of analysis, we used custom-written MATLAB functions to merge behavioral tracking and calcium event data to characterize place cells and spatial engrams (Supplementary Fig. 4). Behavioral data were automatically analyzed and exported from Noldus EthoVision XT15 software. *Behavioral Tracking Analysis*: Mouse speed as a function of time was derived from its XY-location (mouse body center) as a function of time (resolution of 0.04 sec) exported from Noldus software and smoothed with a triangular window (size = 0.3 sec). Mobility was then quantified as the fraction of time that the mouse spent actively exploring (speed > 1 cm/sec) over a 10-minute recording session. Next, an occupancy-time map was created by dividing the arena into a 15-by-15 matrix (i.e. 225 pixels) and computing the duration (in sec) that a mouse spent within each pixel. *Place Cell Analysis*: This stage of analysis is conceptually shown in Supplementary Fig. 4A. First, calcium event timestamps were matched with behavioral tracking timestamps to find the spatial location of the mouse at the beginning of rising phase of each calcium event. Second, calcium events that occurred during periods of inactivity (speed < 1cm/sec) were excluded. Third, for each cell, an amplitude-weighted *calcium event map* was created by duplicating calcium events according to their amplitudes (~1-8 MAD) and distributing them along the track of the mouse, at intervals of 40 msec, over the rising phase of the GCaMP6f fluorescence signal. Fourth, for each cell, a *calcium event rate map* (events/sec; 15-by-15 matrix) was created by dividing the calcium event map by the occupancy-time map,

pixel-by-pixel. Fifth, for each cell, the mutual information (MI) between the calcium event rate map and occupancy-time map for each cell was calculated according to the formulae:

$$I_{pos}(x_i) = \sum_{K\geq 0} P_{k|x_i}\, log_2(\frac{P_{k|x_i}}{P_k})$$

$$MI = \sum_{i=1} P_{x_i}\, I_{pos}(x_i)$$

Where:

- x_i refers to one pixel in the binned arena (in our analysis, the arena is depicted by a 15-by-15 matrix).
- $I_{pos}(x_i)$ is the positional information (in bits) of pixel x_i.
- P_{x_i} is the probability that the mouse occupies pixel x_i, calculated as ($\frac{time\ spent\ in\ x_i}{total\ time\ of\ recording}$).
- P_k is the probability of observing k calcium events
- $P_{k|x_i}$ is the conditional probability of observing k calcium events in pixel x_i, which is equivalent to calcium event rate in pixel x_i.
- MI is the mutual information (in bits per second) between calcium event rate and mouse's spatial location.

(adapted from: (5, 6). Finally, for each cell, a null distribution of time-shuffled MI values was created by 1000 random circular permutations of calcium event timestamps; this null distribution was used to calculate p(MI). To facilitate visualization of place specific firing, rate maps in figures are presented as 50x50 color-coded matrices. *Rate Map (RM) Correlation Analysis*: This analysis is conceptually shown in Supplementary Fig. 4B. First, for each cell, calcium event rate maps were smoothed by a Gaussian filter (radius = 10 cm, sigma = 6 cm) to generate smoothed calcium event rate maps. Second, for each cell, Pearson's correlation coefficient (PCC) was calculated between the vectorized smoothed calcium event rate maps from S1 and S2 (or more broadly, between any two arbitrarily chosen sessions). Third, to detect and account for possible coherent rotation of cells' event rate maps (6), we calculated RM correlation by rotating maps from one session only (either S1 or S2) by 0°, 90°, 180°, and 270°, and the rotation that resulted in maximum mean(RM) was designated as angle of coherent rotation—which usually was 0°. Fourth, for each pair of sessions, the distribution of PCCs from all coactive cells (i.e. those with >5 calcium events during both sessions) was referred to as RM correlation distribution. Finally, for each pair of sessions, a null distribution of RM correlation values was created by 100 random spatial permutations of smoothed calcium rate maps. *Population Vector (PV) Correlation Analysis*: This analysis is conceptually shown in Supplementary Fig. 4C. First, for each pair of sessions, smoothed calcium event rate maps of all coactive cells were organized into two 3-dimensional matrices, thus forming for each pixel a pair of population vectors (PVs). Second, for each pixel, the PCC between the two PVs was calculated, resulting in a PV correlation distribution. Third, to detect and account for coherent rotation, we calculated PV correlation by using the same rotation procedure as in RM correlation computation, and again an angle of coherent rotation based on PV is determined—which again usually was 0° (though RM and PV did not always agree). Fourth, for each pair of sessions, a null distribution of PV correlation values was created by 100 random permutations of PVs for all pixels.

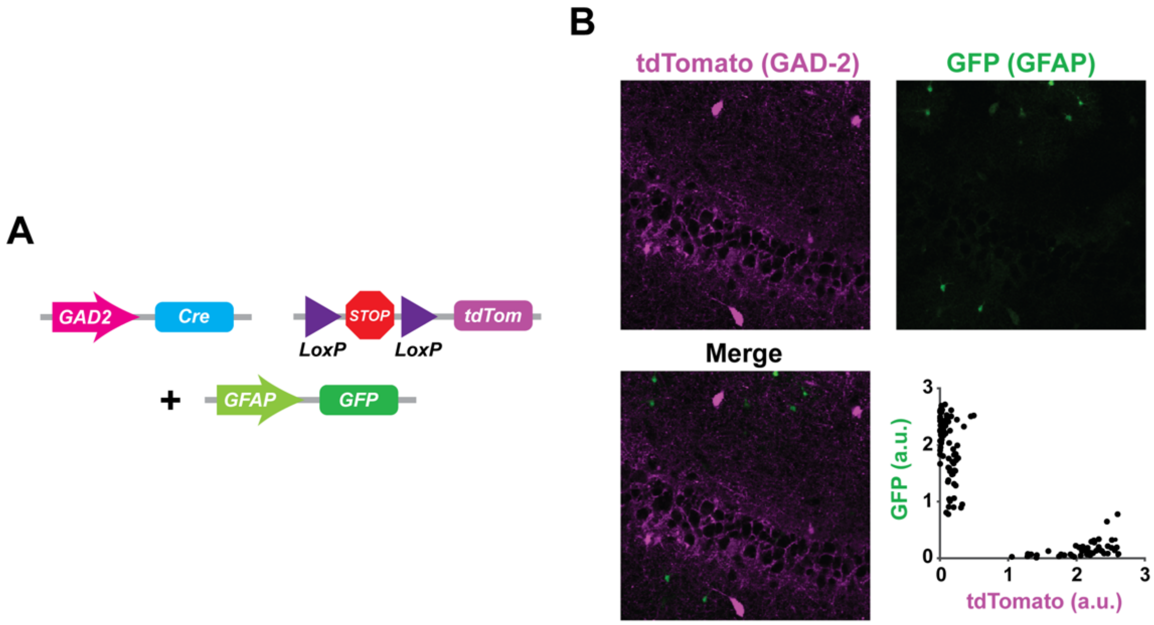

Figure S1. Cre is not expressed in astrocytes in GAD2-Cre mice

(A) Genetic strategy used to test the overlap between Cre recombinase expression in interneurons (Cre-dependent td-Tomato expression) versus astrocytes (GFP driven by the astrocyte-specific GFAP promoter).

(B) Confocal imaging of transgene expression. Fluorescence levels (in a.u.—arbitrary units) of GAD2-Cre-driven td-Tom expression and GFAP-driven GFP expression were highly segregated, demonstrating a lack of GAD2-driven Cre recombinase in astrocytes.

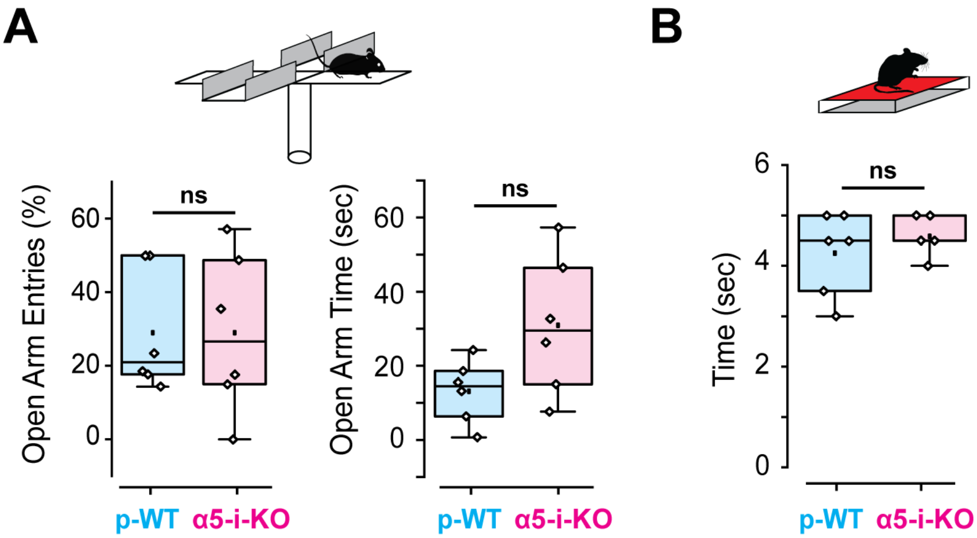

Figure S2. Baseline behavioral characteristics of p-WT vs. α5-i-KO mice

(A) There were no differences in elevated plus maze open arm entries ($t(9.31) = 0.000066$, $p = 0.99$, Welch's t test), or open arm entry time ($t(6.9) = 2.1$, $p = 0.073$, Welch's t test) between the two genotypes ($n = 6$ for each), indicating a lack of difference in anxiety.

(B) Latency to paw withdrawal was not different between the two genotypes ($n = 6$ for each; $t(7.0) = 0.91$, $p = 0.40$, Welch's t test), indicating no difference in pain sensitivity. For both parts, each diamond symbol represents one mouse, and data are graphed in quartiles with standard deviation.

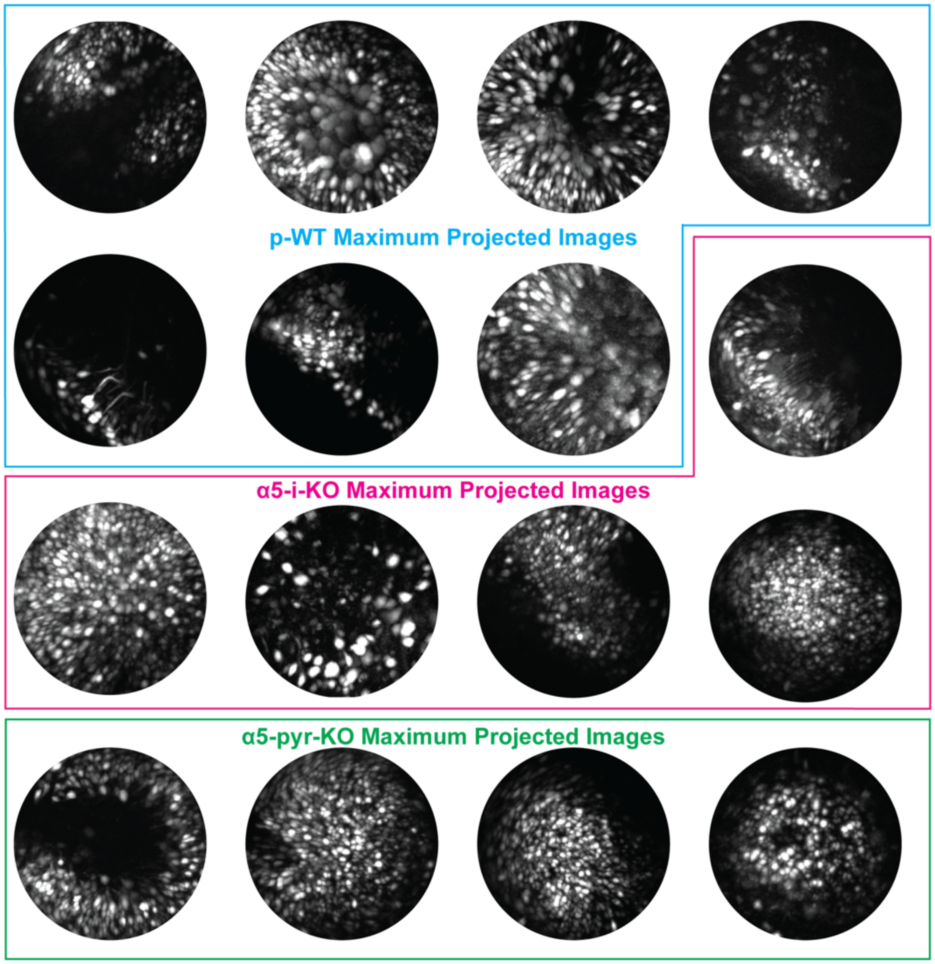

Figure S3. Maximum projected images from Ca^{2+} imaging recordings

A range of ~80 to ~800 cells were programmatically captured from CNMF-E algorithm in the first stage of analysis.

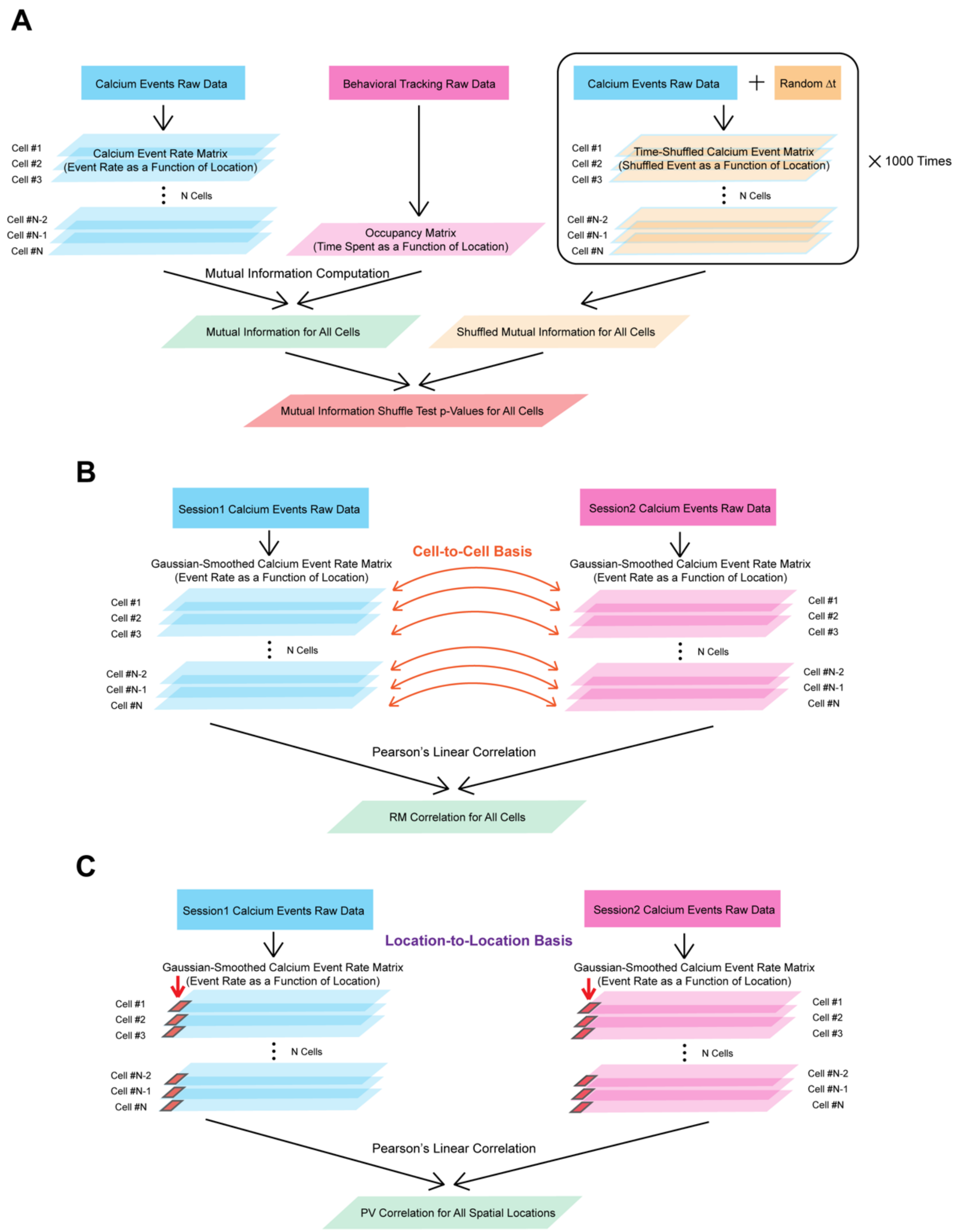

Figure S4. Analysis workflow for place cells and spatial engrams

(A) Calculation of place-specific firing by mutual information (cells with p(MI)<0.05 are classified as place cells).

(B) Calculation of cell-based rate map (RM) correlation for both place and non-place cells.

(C) Calculation of position-based population vector (PV) correlation for both place and non-place cells.

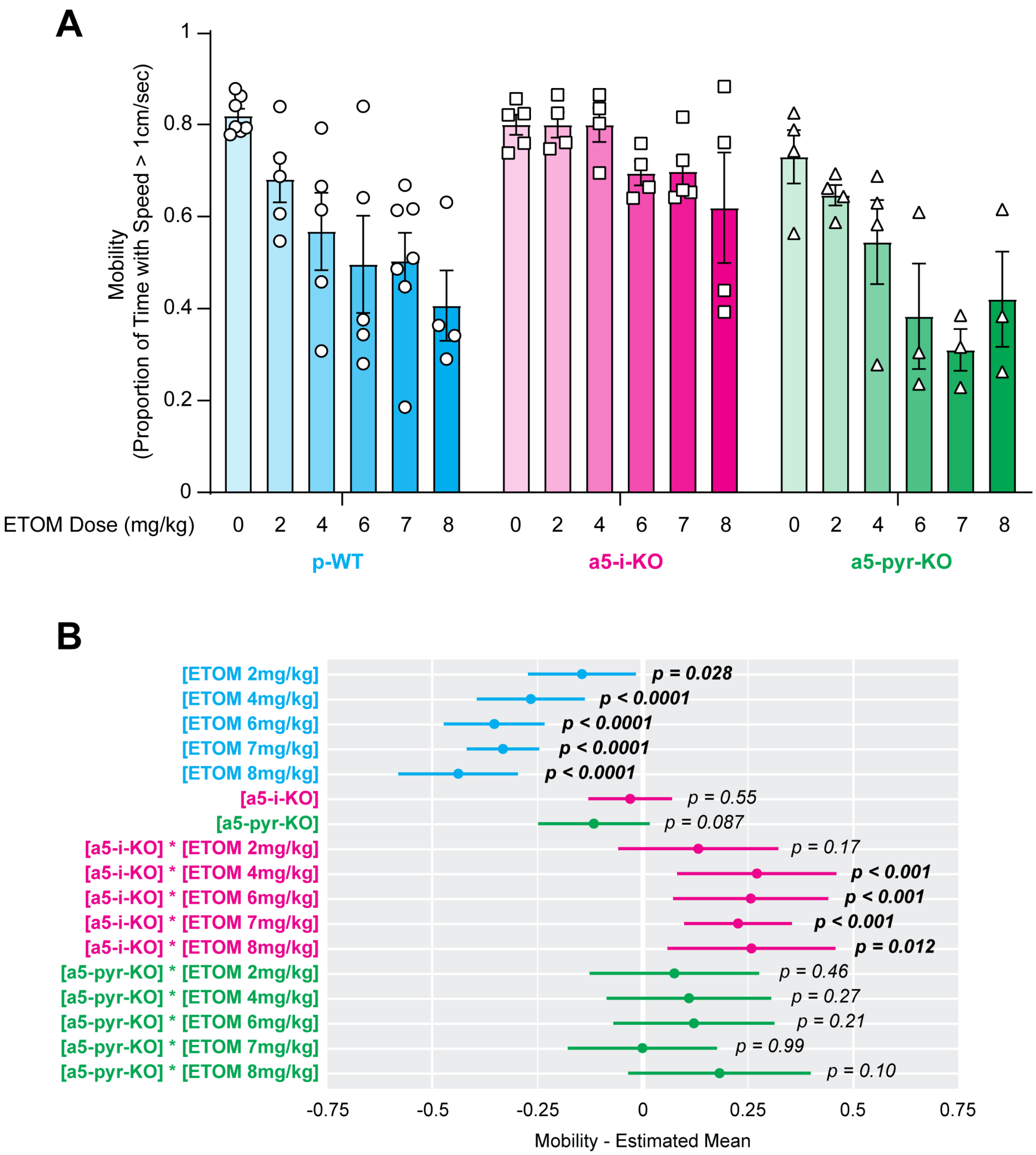

Figure S5. Dose-dependent effect of etomidate on mobility.

(A) Fraction of time mice actively explored (± sem) as a function of etomidate dose. Each point represents the mean mobility throughout a 10-min session, for all sessions under a given condition, for each mouse.

(B) A linear mixed effects model was used to evaluate drug effect (first five rows), genotype effect (next two rows), and genotype-drug interactions for mean event rate (next ten rows), using p-WT [genotype] and saline [drug] as reference levels. Etomidate reduced mobility at all doses in p-WT mice. No genotype effect was observed in the reference drug (saline) condition, indicating that elimination of α5-$GABA_A$Rs from interneurons or pyramidal neurons did not alter intrinsic activity. Significant genotype-drug interactions were observed at etomidate doses of 4, 6, 7, and 8 mg/kg for α5-i-KO but not α5-pyr-KO mice, indicating that α5-i-KO mice partially resisted the sedative effect of etomidate.

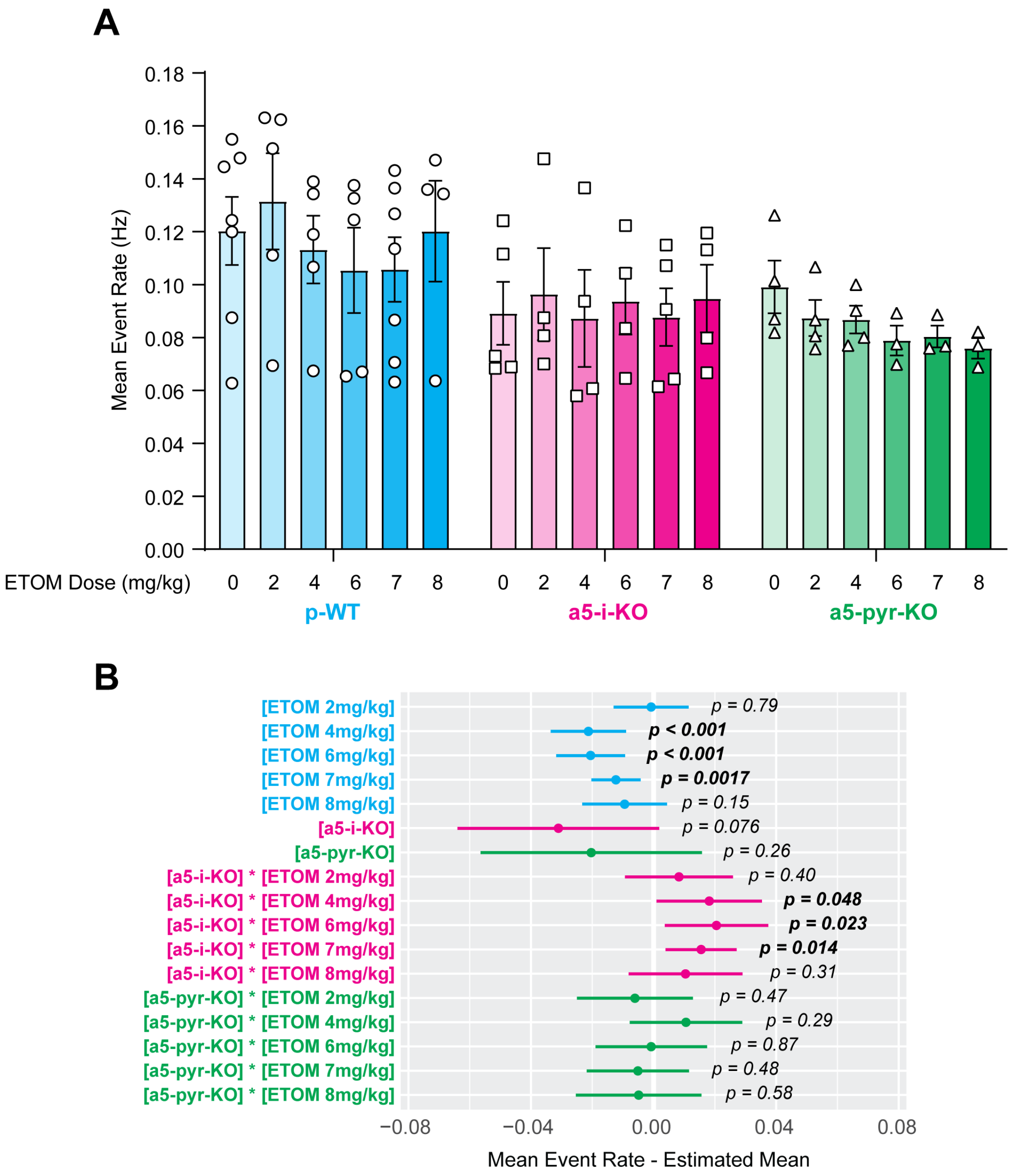

Figure S6. Dose-dependent effect of etomidate on mean Ca^{2+} event rate.

(A) Mean event rate (± sem) as a function of etomidate dose for all three genotypes. Each point represents the mean event rate of all cells in all sessions under a given condition for each mouse.

(B) A linear mixed effects model was used to evaluate drug effect (first five rows), genotype effect (next two rows), and genotype-drug interactions for mean event rates (next ten rows), using p-WT [genotype] and saline [drug] as reference levels. Though not visually obvious in part (A), slight (-0.01 to -0.02 Hz) but statistically significant reductions were seen in mean even rate at etomidate doses of 4, 6, and 7mg/kg in p-WT mice. No genotype effect was observed in the reference drug (saline) condition, indicating that elimination of α5-GABA$_A$Rs from either interneurons or pyramidal neurons did not influence cellular activity as reported by Ca^{2+} events. Interestingly, we observed complete resistance to the event rate-suppressing effect of etomidate at doses of 4, 6, and 7mg/kg in α5-i-KO mice, but not in α5-pyr-KO mice.

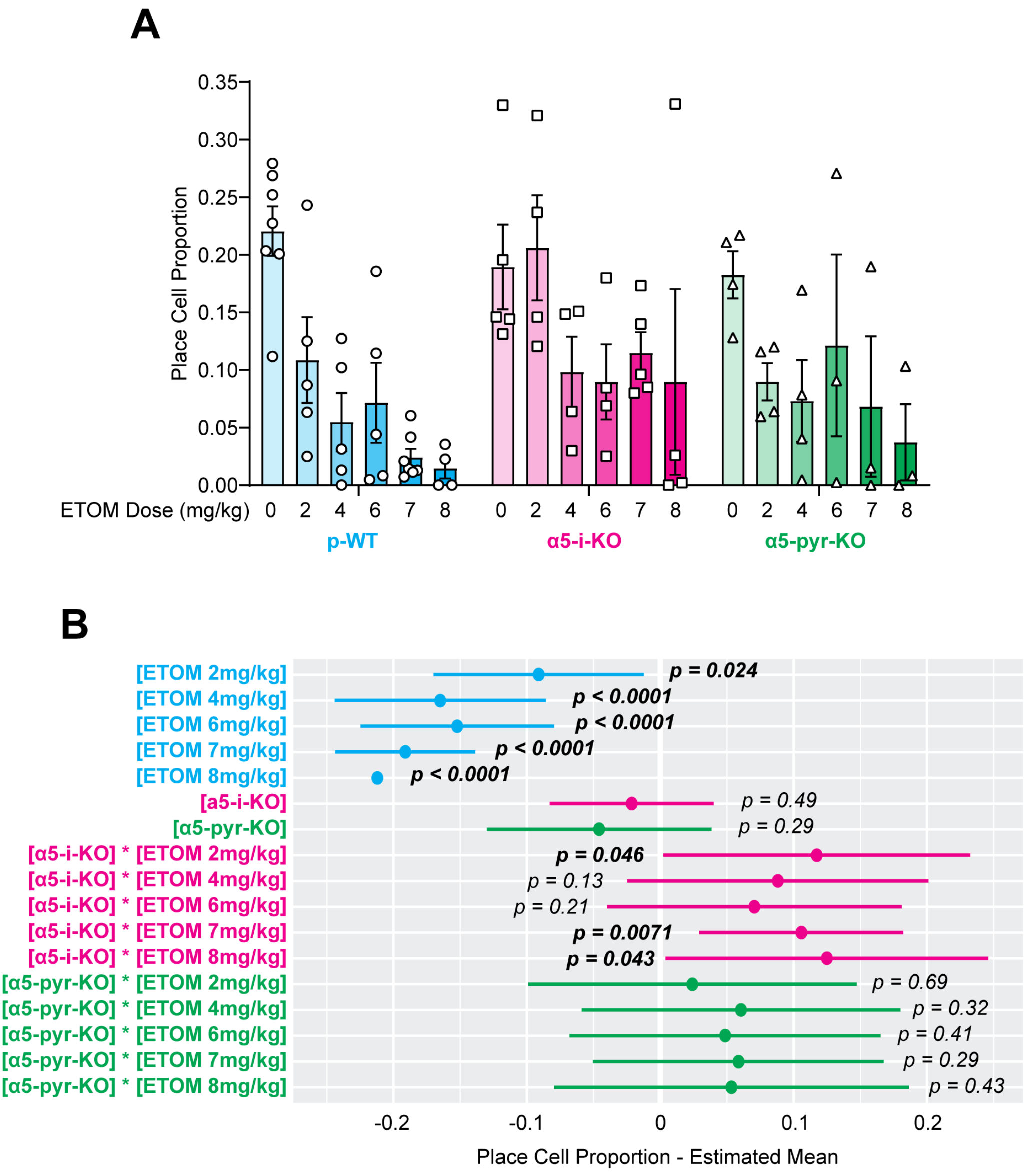

Figure S7. Dose-dependent effect of etomidate on place cells

(A) Mean place cell proportion (± sem) as a function of etomidate dose. Each point represents the mean proportion of all active cells in a session that qualified as bona-fide place cells (p(MI)<0.05) for all sessions under a given condition for each mouse.

(B) A linear mixed effects model was used to evaluate drug effect (first five rows), genotype effect (next two rows), and genotype-drug interactions for mean event rate (next ten rows), using p-WT [genotype] and saline [drug] as reference levels. Etomidate strongly suppressed place cell formation at all doses in p-WT mice. No genotype effect was observed in the reference drug (saline) condition, indicating that elimination of α5-$GABA_A$Rs from interneurons or pyramidal neurons did not influence place cell formation under control conditions. Significant genotype-drug interactions were observed at etomidate doses of 2, 7, and 8 mg/kg for α5-i-KO but not α5-pyr-KO mice, implicating interneuronal α5-$GABA_A$Rs as essential targets by which etomidate suppresses spatially modulated firing of pyramidal neurons.

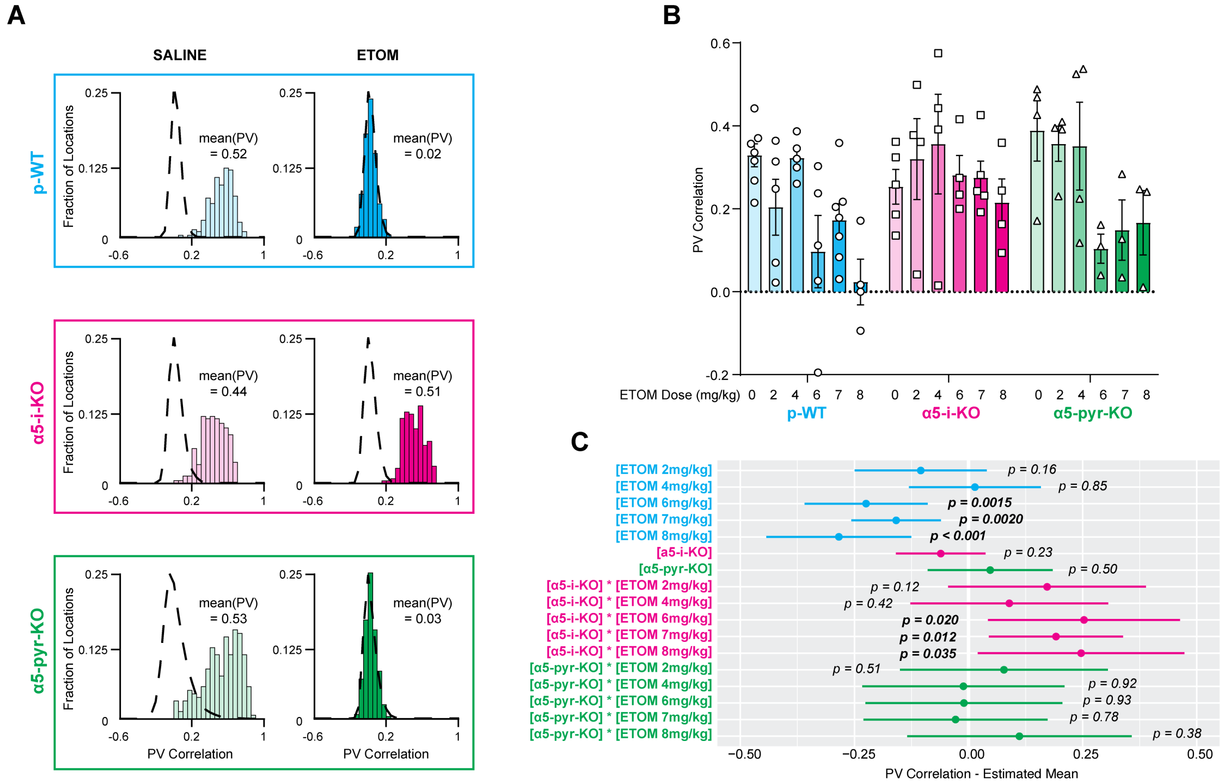

Figure S8. PV correlation analysis

(A) Distributions of PV correlations from six paired recording sessions in p-WT (top), α5-i-KO (middle), and α5-pyr-KO (bottom) mice that were administered saline (left) or 7mg/kg etomidate (right). Left: under control conditions, distributions fell substantially to the right of the location-shuffled PV null distributions (dashed lines) in all three genotypes, revealing the presence of stable spatial engrams. Right: etomidate caused the distributions to be shifted toward the null distributions in p-WT and α5-pyr-KO mice but not in α5-i-KO mice.

(B) Mean PV_{corr} (± sem) as a function of etomidate dose. Each point represents the mean PV_{corr} value for all cells active in both of a pair of matched sessions, for all sessions under a given condition for each mouse.

(C) A linear mixed effects model was used to evaluate drug effect (first five rows), genotype effect (next two rows), and genotype-drug interactions for mean event rate (next ten rows), using p-WT [genotype] and saline [drug] as reference levels. Etomidate reduced PV_{corr} at doses of 6, 7, and 8mg/kg in p-WT mice. No genotype effect was observed in the reference drug (saline) condition, indicating that elimination of α5-$GABA_A$Rs from interneurons or pyramidal neurons did not influence PV_{corr} under control conditions. Significant genotype-drug interactions were observed at etomidate doses of 6, 7, and 8 mg/kg for α5-i-KO but not α5-pyr-KO mice, implicating interneuronal α5-$GABA_A$Rs as essential targets by which etomidate suppresses spatial engrams as reported by PV_{corr}.

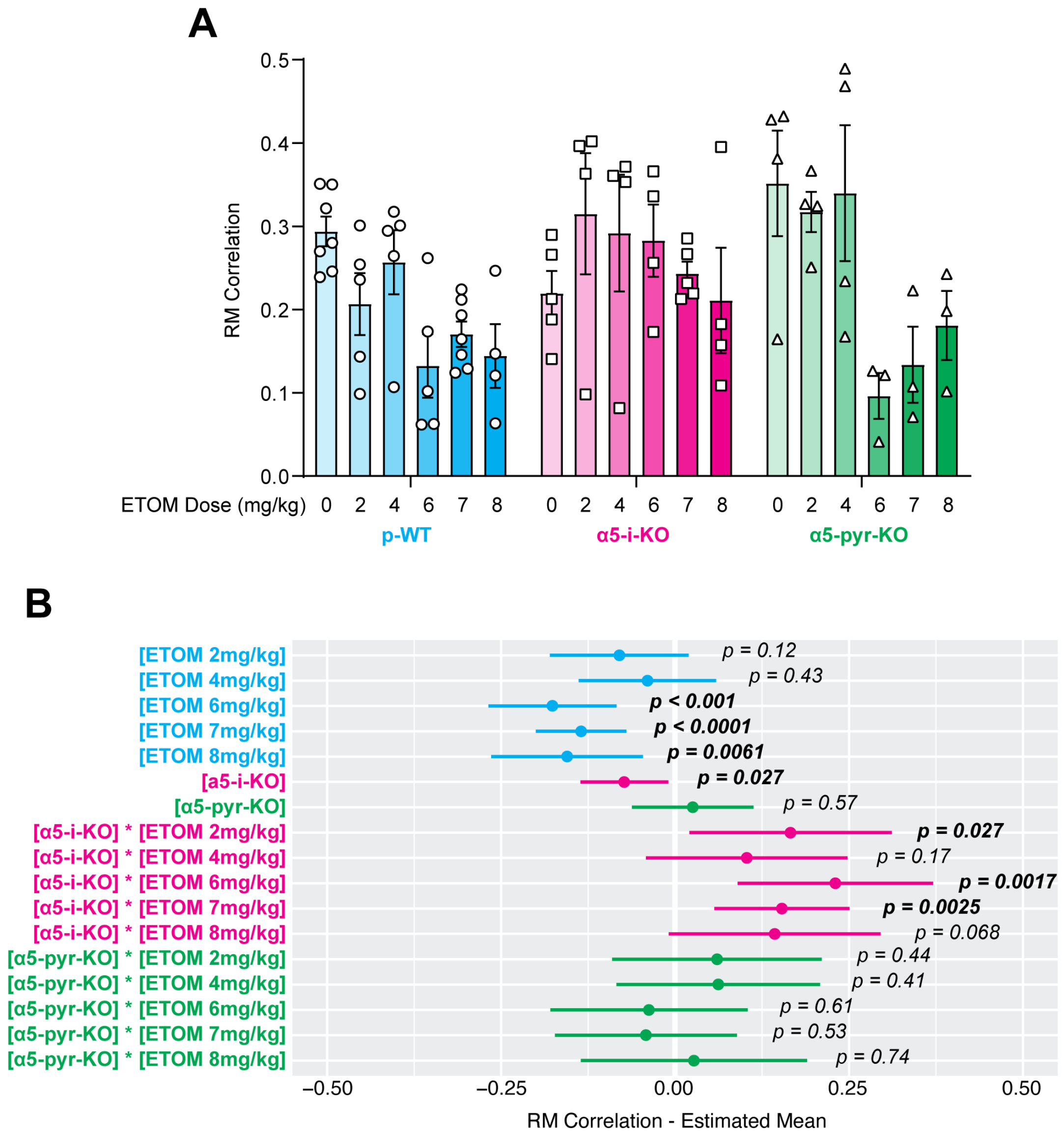

Figure S9. Dose-dependent effect of etomidate on RM correlation

(A) Mean RM_{corr} ($\pm$ sem) as a function of etomidate dose. Each point represents the mean RM_{corr} value for all cells active in both of a pair of matched sessions, for all sessions under a given condition for each mouse.

(B) A linear mixed effects model was used to evaluate drug effect (first five rows), genotype effect (next two rows), and genotype-drug interactions for mean event rate (next ten rows), using p-WT [genotype] and saline [drug] as reference levels. Etomidate reduced RM_{corr} at doses of 6, 7, and 8mg/kg in p-WT mice. A significant genotype effect in α5-i-KO mice in the reference drug (saline) condition indicates that α5-$GABA_A$Rs in interneurons exert a physiological memory-promoting influence. Significant genotype-drug interactions were observed at etomidate doses of 6, 7, and 8 mg/kg for α5-i-KO but not α5-pyr-KO mice, implicating interneuronal α5-$GABA_A$Rs as essential targets by which etomidate suppresses spatial engrams as reported by RM_{corr}.

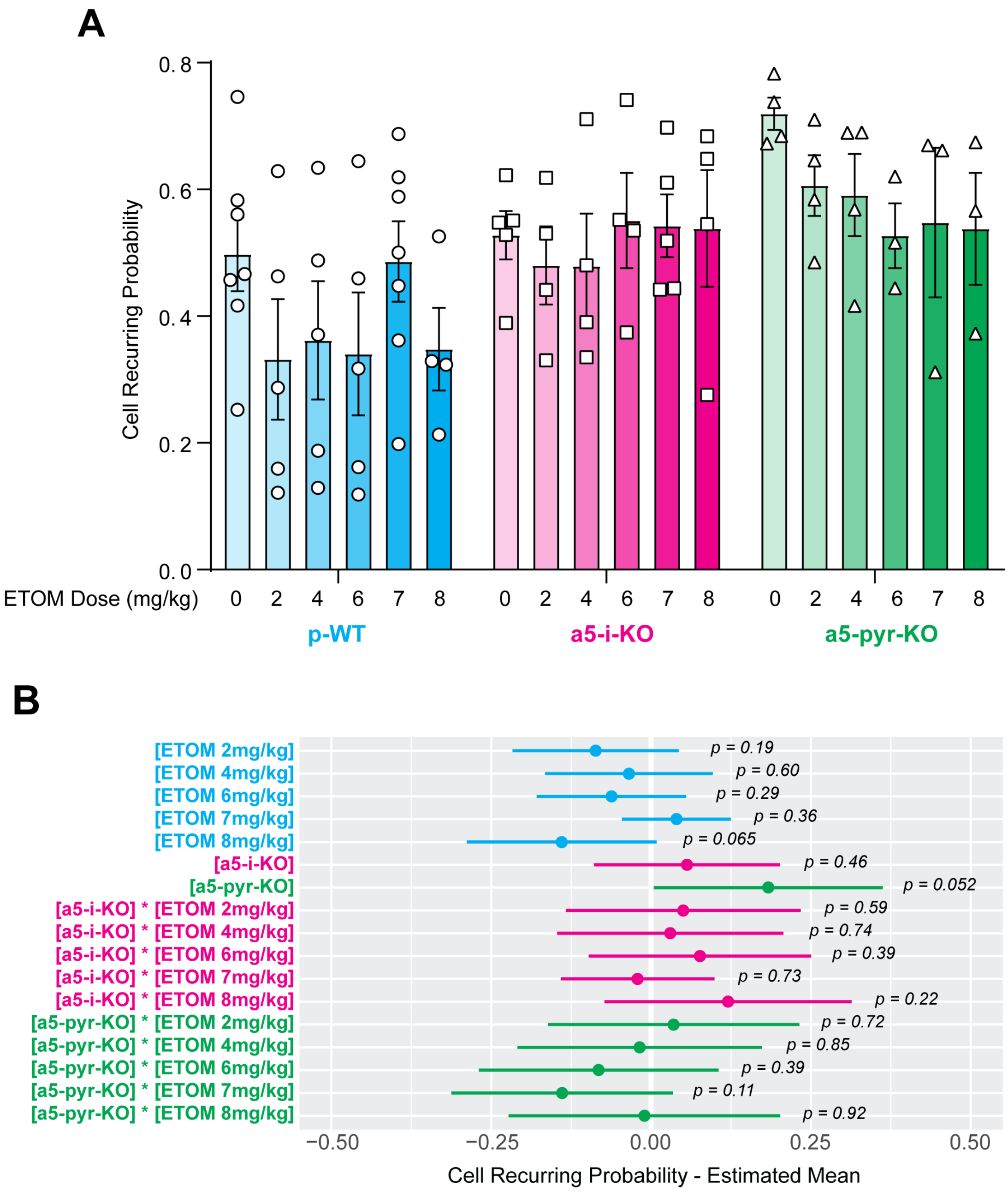

Figure S10. Lack of effect of etomidate on cell recurrence probability

(A) Mean recurring probability (± sem) as a function of etomidate dose. Each point represents the mean recurring probability for all cells active in the AM session for all pairs of matched sessions under a given condition for each mouse.

(B) A linear mixed effects model was used to evaluate drug effect (first five rows), genotype effect (next two rows), and genotype-drug interactions for mean event rate (next ten rows), using p-WT [genotype] and saline [drug] as reference levels. Etomidate did not have any effect on recurring probability at any dose in any genotype.

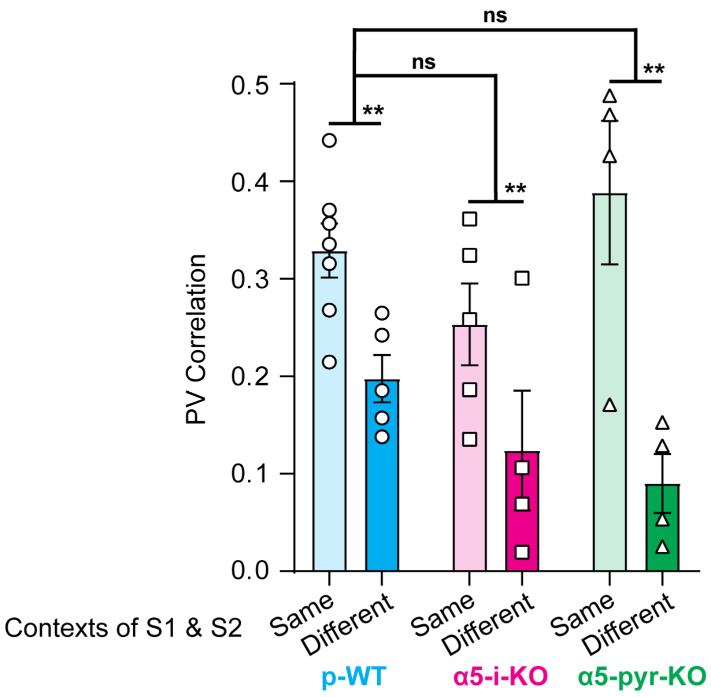

Figure S11. Summary of different-context experiments for PV correlation. Similar to RM correlation, in all genotypes, changing contextual cues produced a significant reduction in PV correlation. No genotype-experimental condition interactions were observed, indicating that all three genotypes distinguished contexts equally well.

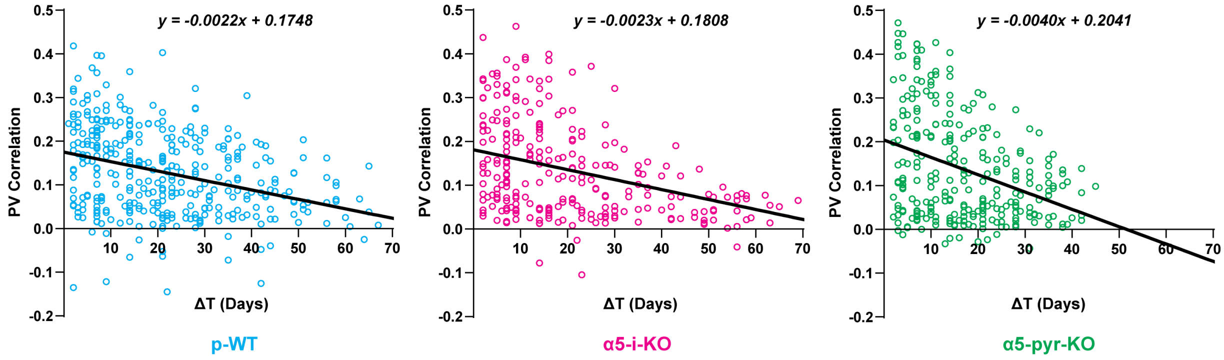

Figure S12. Cross-day different contexts experiments analyzed using PV correlation. The conclusions and interpretations were essential identical to that of the RM correlation.

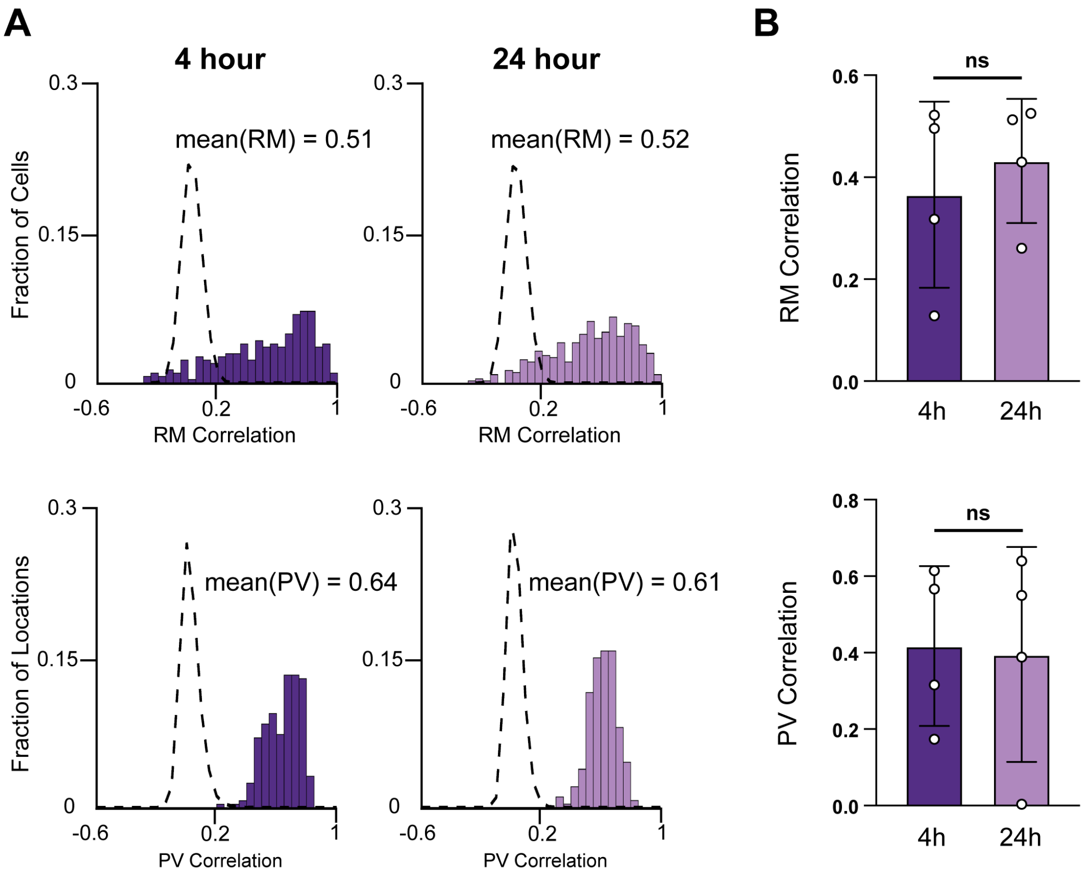

Figure S13. 4hr vs 24hr spatial engram stability experiments conducted in a separate cohort of four C57BL/6J mice

(A) Representative RM and PV correlation distributions when same-context experiments were conducted over 4hrs vs. 24hrs. All four distributions fall significantly outside of their corresponding shuffled null distributions, indicating formation of stable spatial engrams. More importantly, spatial engram stability was essentially identical over 4hrs vs. 24hrs.

(B) Summary of 4hr vs. 24hr experiments, where RM and PV correlations show approximately equal strengths.

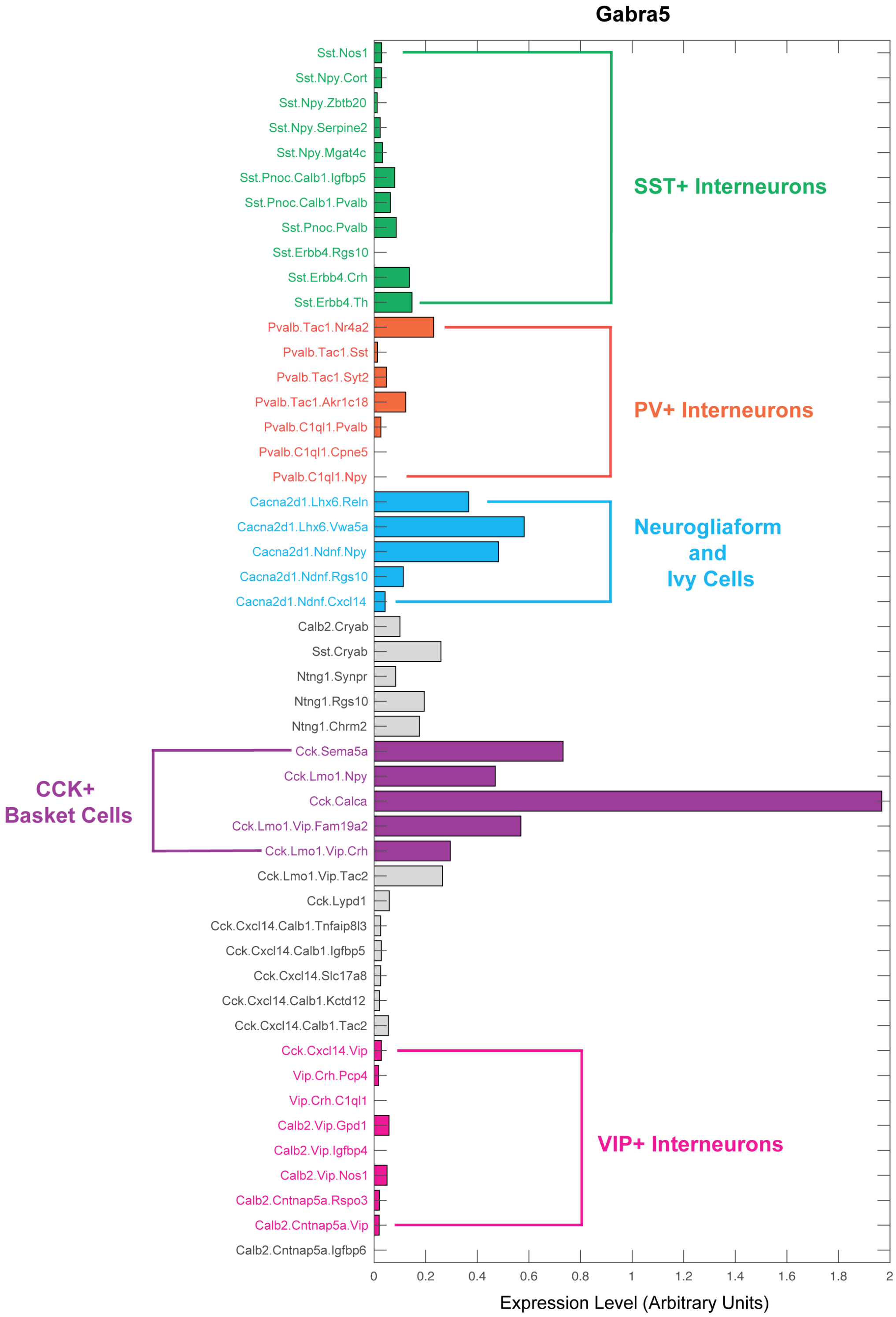

Figure S14. Transcriptome profile of Gabra5 mRNA in hippocampal CA1 interneurons. Cell classes are named according to the publication from which the data were obtained (7). Expression levels were extracted using software provided by the author, together with custom-written MATLAB codes.

References

1. P. Zhou *et al.*, Efficient and accurate extraction of in vivo calcium signals from microendoscopic video data. *Elife* **7** (2018).
2. J. Friedrich, P. Zhou, L. Paninski, Fast online deconvolution of calcium imaging data. *PLoS Comput Biol* **13**, e1005423 (2017).
3. T. W. Chen *et al.*, Ultrasensitive fluorescent proteins for imaging neuronal activity. *Nature* **499**, 295-300 (2013).
4. L. Sheintuch *et al.*, Tracking the Same Neurons across Multiple Days in Ca(2+) Imaging Data. *Cell Rep* **21**, 1102-1115 (2017).
5. A. V. Olypher, P. Lánský, R. U. Muller, A. A. Fenton, Quantifying location-specific information in the discharge of rat hippocampal place cells. *J. Neurosci. Methods* **127**, 123-135 (2003).
6. N. R. Kinsky, D. W. Sullivan, W. Mau, M. E. Hasselmo, H. B. Eichenbaum, Hippocampal Place Fields Maintain a Coherent and Flexible Map across Long Timescales. *Curr. Biol.* **28**, 3578-3588 e3576 (2018).
7. K. D. Harris *et al.*, Classes and continua of hippocampal CA1 inhibitory neurons revealed by single-cell transcriptomics. *PLoS Biol* **16**, e2006387 (2018).
